# Detection of DNA Sequences Refractory to PCR Amplification Using a Biophysical SERRS Assay (Surface Enhanced Resonant Raman Spectroscopy)

**DOI:** 10.1371/journal.pone.0114148

**Published:** 2014-12-12

**Authors:** Cécile Feuillie, Maxime M. Merheb, Benjamin Gillet, Gilles Montagnac, Isabelle Daniel, Catherine Hänni

**Affiliations:** 1 Laboratoire de Géologie de Lyon – Terre, Planètes, Environnement, UMR 5276, ENS de Lyon, Université Lyon 1, CNRS, Ecole Normale Supérieure de Lyon, 46 allée d′Italie, 69364 Lyon Cedex 07, France; 2 Institut de Génomique Fonctionnelle de Lyon, UMR 5242, Université Lyon 1, CNRS, Ecole Normale Supérieure de Lyon, 46 allée d′Italie, 69364 Lyon Cedex 07, France; 3 Plateforme nationale de Paléogénétique PALGENE, CNRS, Ecole Normale Supérieure de Lyon, 46 allée d′Italie, 69364 Lyon Cedex 07, France; Northeastern University, United States of America

## Abstract

The analysis of ancient or processed DNA samples is often a great challenge, because traditional Polymerase Chain Reaction – based amplification is impeded by DNA damage. Blocking lesions such as abasic sites are known to block the bypass of DNA polymerases, thus stopping primer elongation. In the present work, we applied the SERRS-hybridization assay, a fully non-enzymatic method, to the detection of DNA refractory to PCR amplification. This method combines specific hybridization with detection by Surface Enhanced Resonant Raman Scattering (SERRS). It allows the detection of a series of double-stranded DNA molecules containing a varying number of abasic sites on both strands, when PCR failed to detect the most degraded sequences. Our SERRS approach can quickly detect DNA molecules without any need for DNA repair. This assay could be applied as a pre-requisite analysis prior to enzymatic reparation or amplification. A whole new set of samples, both forensic and archaeological, could then deliver information that was not yet available due to a high degree of DNA damage.

## Introduction

Polymerase Chain Reaction (PCR) based methods allow a rapid detection and identification of DNA sequences by amplifying minute amounts of DNA. By targeting highly specific regions of mitochondrial DNA for instance, it is possible to identify to the species-level meat [1], [2], highly processed samples [3], forensic [4] or archaeological remains [5]. This way, bones may be identified despite a lack of morphological criteria [5], [6]. Nevertheless, when working with archaeological or forensic samples, PCR amplification often fails. For instance, Höss et al. [7] studied 35 samples of Late Pleistocene sloths, among which only 2 lead to successful PCR amplification. Environmental conditions are key parameters for the preservation of amplifiable DNA; over 62% of permafrost samples lead to successful enzymatic amplification, whereas samples from hot and arid climates merely give a 2 to 4% success rate [8]. PCR failure is directly linked to the degradation content of the DNA template [9]. DNA undergoes severe modification after death [10], first from endonucleases and microorganisms, then by hydrolysis and oxidation reactions [11], [12]. Several types of DNA damage called blocking lesions stop the bypass of polymerases, preventing primer elongation: oxidized pyrimidines, also referred to as hydantoins, cross-links and abasic sites [9], [13]–[17]. In abasic sites, neither a purine nor a pyrimidine base is present. Höss et al. [13] have shown that hydantoins were present in substantially higher amount in samples that inhibited PCR amplification. Sikorsky *et al.* [15], [16] report a decrease of the mean PCR efficiency of up to 98.2% when one abasic site is present in the DNA template. It is therefore possible to obtain a false-negative PCR amplification if the DNA template includes blocking lesions. Enzymatic amplification can also lead to false-positive results by producing non-authentic sequences. First, chimeric sequences may be obtained by jumping PCR [18], where partially elongated primers jump from one degraded DNA template to another, leading to recombinant sequences. Second, some DNA damage called miscoding lesions do not stop the elongation but lead to errors in the final copied sequence. For instance, the loss of an amine group on the cytosine, leading to uracil residue, would be the main cause of miscoding errors [19]. The presence of abasic sites in DNA also reduces the fidelity of *Taq* polymerase, leading to deletions and miscoding [15], [16], [20]. Brotherton et al. [21] proposed a method called SPEX (single primer extension) that successfully produces accurate DNA sequences from damaged DNA templates. However, despite its huge potential SPEX hasn't been widely applied because of its tedious protocol [22]. Reparative enzymatic techniques have also been proposed; for instance, Briggs et al. [23] combine two enzymes, the uracil-N-glycosylase and the endonuclease VIII, to both remove the uracil residues and eliminate the resulting abasic sites. Nevertheless, there is no direct way to check for the presence of target DNA prior to the repair procedures that are time-consuming and expensive. Another advance involved the development of new polymerases that can bypass lesions such as the abasic sites and the hydantoins [24]–[26], [9]. However, these damage-tolerant enzymes could not guarantee a significantly higher amplification [24], [25], [9], and sometimes showed a lower fidelity in the case of ancient DNA analysis [9], [24]. A truly non-enzymatic DNA detection method would be very useful to quickly and specifically detect DNA sequences that might be refractory to enzymatic amplification, especially in degraded and processed samples.

Non-enzymatic methods of detection have been developed for single-stranded DNA, using for instance fluorescence [27], [28], scanometric detection combined to silver enhancement [29], [30] or Surface Enhanced Resonance Raman Scattering (SERRS) [31]–[34]. However the non-enzymatic detection of double-stranded DNA is a greater challenge because of the extreme affinity of the target strand for its complementary strand. Hill et al. [35] detected double-stranded DNA down to a detection limit of 2.5 fmol/L with a non-enzymatic assay based on hybridization with functionalized nanoparticles, silver enhancement, and scanometric detection. Despite its high efficiency, this assay requires long multiple steps. To circumvent this, we have recently developed a SERRS-hybridization assay, complementary to Hill's method, that allows for the quick and specific detection of double-stranded DNA sequences in ca. 2 hours only [36]. This fully non-enzymatic SERRS-hybridization assay enables quantification of multiple double-stranded DNA sequences from closely related species in a single measurement with only 15% uncertainty even for target's length below 100 bp. Being non-enzymatic, this assay should not be impeded by the presence of blocking lesions or PCR inhibitors and therefore has the potential to detect degraded double-stranded DNA that might be reluctant to enzymatic amplification.

In the present study we evaluated the potential of this newly developed SERRS-hybridization assay to detect degraded double-stranded DNA refractory to enzymatic amplification by PCR. We studied a series of synthetic double-stranded DNA molecules that contain analogs of abasic sites, as an example of blocking lesion. These molecules were analyzed both by a classical PCR reaction and by the new SERRS-hybridization assay. The latter biophysical assay allowed us to detect all molecules, whereas PCR failed to detect the most degraded ones. This leads us to propose that this assay could be applied as a rapid and convenient pre-requisite analysis prior to enzymatic reparation or amplification. A whole new set of samples, both forensic and archaeological, could then deliver information that was unavailable until now due to a high degree of DNA damage.

## Material and Methods

### Reagents

All reagents were analytical grade. Tetrahydrochloride spermine (Fluka, #85610), Polyoxyethylenesorbitan monolaurate (Tween20, #P1379) and silver nitrate 99.999% (#S8157) were purchased from Sigma-Aldrich. 1% trisodium citrate (#S1804) was from Fisher. Ultra pure 20xSSC Buffer (Gibco, #15557-044), Streptavidin-coated magnetic microbeads (Dynal, Dynabeads MyOne Streptavidin C1, #650-02, 10 mg.ml^−1^, 2 ml, 7–12×10^9^ beads) and the matching DynaMag-2 magnetic separator (Dynal, #123-21D) were purchased from Life Technologies.

### Target DNA sequences

All sequences were purchased from Eurogentec and are listed in Table 1. We studied 11 double-stranded DNA molecules containing a varying number of abasic sites between none and nine, distributed on both strands. The original non-degraded molecule is a 139 bp sequence of mitochondrial DNA (12S rRNA gene) of *Rupicapra rupicapra* (Chamois). The analog of abasic sites used in the present study is tetrahydrofuran (THF), inserted by the manufacturer on both strands during their synthesis. The 3′-5′ and 5′-3′ strands contained 0 to 5 and 0 to 4 analogs of abasic sites, respectively. All double-stranded molecules were obtained from the specific hybridization of their complementary strands before the experiment. Solutions of each complementary strand at a concentration of 2×10^−7^ mol.l^−1^ were mixed and left at room temperature for at least 60 hours, leading to a solution of double-stranded DNA at a concentration of 10^−7^ mol.l^−1^.

**Table 1:** Nucleic sequences used in this study.

Name	Sequence	AS
**N_5′_**	GCCATGAAGCACGCACACACCGCCCGTCACCCTCCTCAAGTGAA**T**ACAGGACA**C**TCAAAACC**TA**TTTAAAC**A**C**A**CCAATC**A**CACAAGAGGAGACAAGTCGTAACAAGGTAAGCATACTGGAAAGTGTGCTTGGACAAAC	0
	*Bases ranging from 1 to 139*	
**I_5′_**	.........................................................................*.................................................................	1
	*74*	
**II_5′_**	...............................................................*.........*.................................................................	2
	*64 74*	
**V_5'_**	............................................*........*........*........*........*..........................................................	5
	*45 54 63 72 81*	
**Cap_3_**_′_	---------------------------------GGAGTTCACTTATGTCCTGTG-------------------------------------------------------------------------------------	-
**Det_3_**_′_	-----------------------------------------------------------------------------------GTTCTCCTCTGTTCAGCATTGT----------------------------------	-
**Rup_Rev_3_**_′_	----------------------------------------------------------------------------------------------------------------------CCTTTCACACGAACCTGT---	-
**N_3'_**	CGGTACTTCGTGCGTGTGTGGCGGGCAGTGGGAGGAGTTCACTTATG**TC**CTGTGAGT**T**TTGGATAA**A**TTTGTGTG**G**TTAGTGTGTTCTCCTCTGTTCAGCATTGTTCCATTCGTATGACCTTTCACACGAACCTGTTTG	0
**I_3'_**	...............................................*...........................................................................................	1
	*48*	
**IV_3'_**	................................................*........*........*........*...............................................................	4
	*49 58 67 76*	
**Block1_5_**_′_	—CATGAAGCACGCACACACCGCCCGTCACCCT----------------------------------------------------------------------------------------------------------	-
**Block2_5_**_′_	------------------------------------------------------TCAAAACCTATTTAAACACACCAATCACA--------------------------------------------------------	-
**Block3_5_**_′_	---------------------------------------------------------------------------------------------------------AGGTAAGCATACTGGAAAGTGTGCTTGGACA---	-
**Rup_For_5_**_′_	—CATGAAGCACGCACACACCGC--------------------------------------------------------------------------------------------------------------------	-

5′ and 3′ subscripts indicate sequences in the 5′-3′ and 3′-5′ orientations, respectively. Sequences are aligned, and abasic sites (AS) are labeled in red with their positions. PCR primers Rup_For_5′_ and Rup_Rev_3′_ and their alignment to target sequences are also represented. SERRS capture probe, detection probe and 3 blockers are labeled Cap_3′_, Det_3′_, Block1_5′_, Block2_5′_ and Block3_5′_, respectively.

### Target DNA nomenclature

The composition of the synthetic DNA molecules used in this study including the location of abasic sites is presented in Table 1. Four strands oriented 5' to 3′ have been considered and contain 0, 1, 2 or 5 abasic sites, respectively. Complementary 3' to 5' strands carry 0, 1 or 4 abasic sites, respectively. Hereafter, the molecules analyzed in this study are labeled according to their content in abasic sites written as a roman numeral (R) from I to V. All molecules are therefore named in the form R_5′_/R_3′_, where R_5′_ and R_3′_ represent the 5′-3′ and the 3′-5′ strand, respectively. N stands for no abasic sites and indicates that the strand is non-degraded. For instance, II_5′_/N_3′_ corresponds to a 5′-3′ strand containing 2 abasic sites hybridized to a 3′-5′ strand free of abasic sites. Eleven combinations of double-stranded DNA R_5′_/R_3′_ were analyzed in this study both by PCR and by the SERRS-hybridization assay and are listed in Table 2.

**Table 2:** Combination of double-stranded R_5′_/R_3′_ molecules investigated in this study.

Name	Abasic sites on R_5′_	Abasic sites on R_3′_
**N_5′_/N_3′_**	0	0
**N_5′_/I_3′_**	0	1
**II_5′_/N_3′_**	2	0
**N_5′_/IV_3′_**	0	4
**V_5′_/N_3′_**	5	0
**I_5′_/I_3′_**	1	1
**II_5′_/I_3′_**	2	1
**I_5′_/IV_3′_**	1	4
**V_5′_/I_3′_**	5	1
**II_5′_/IV_3′_**	2	4
**V_5′_/IV_3′_**	5	4

N_5′_/N_3′_ is the original non-degraded molecule.

### SERRS hybridization assay

**Figure 1:**
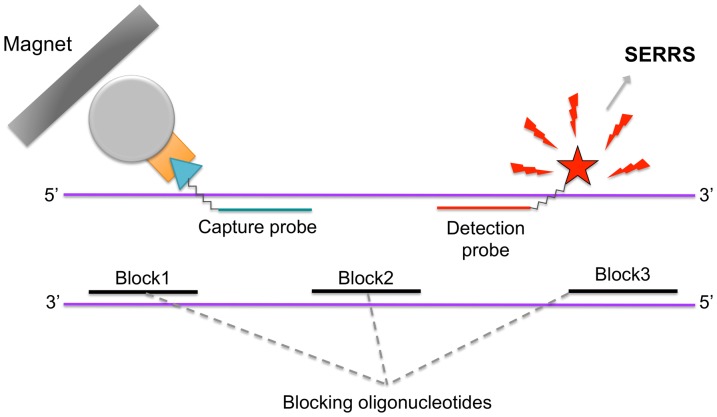
SERRS-hybridization assay principle of double-stranded DNA detection. One strand is specifically hybridized to 2 probes, a biotin-labeled capture probe and a R6G-labeled detection probe. The second strand is specifically hybridized to three oligonucleotides which block the rehybridization to its complementary strand.

The principle of the SERRS hybridization assay is summarized in Figure 1. It takes advantage of the great versatility of the SERRS-hybridization assay recently developed for the specific detection of double-stranded DNA [36], and optimized for the detection of the non-degraded target sequence considered in this study, *i.e.* a 139 bp DNA sequence of mitochondrial DNA (12S rRNA) of *Rupicapra rupicapra* (Chamois). After denaturation of double-stranded DNA target, one strand hybridizes specifically to both a capture probe and a detection probe. The capture probe is a biotinylated oligonucleotide that hybridizes to the strand at its 5′ end and allows the immobilization of the complex on streptavidin-coated magnetic microbeads. The detection probe is a Rhodamine 6G (R6G) labeled oligonucleotide, which hybridizes at the 3′ end of the strand and will subsequently be detected by SERRS. The rapid rehybridization of the target DNA to its complementary strand is prevented by using small oligonucleotides of 30, 29 and 31 bases hereafter called blockers, which hybridize specifically to the complementary strand (Figure 1). The three blockers are added 10^3^ times in excess compared to target DNA concentration and fully prevent rehybridization, thus leaving the target strand available for hybridization with both probes [36]. After this first step, all unbound compounds are washed off and the detection probes are recovered after a thermal dissociation step. They are subsequently detected by SERRS. The whole analysis is completed in 2 hours. Experimental details are provided in the following sections.

#### Hybridization of target DNA with blocking oligonucleotides, capture and detection probes

Hybridization was performed in a single step in a 4xSSC, Tween 20 (0.5%) buffer. A SERRS 22-mer oligonucleotide labeled with one molecule of Rhodamine 6G – R6G (detection probe), and a biotin labeled 21-mer oligonucleotide (capture probe) are hybridized to one strand of the target molecule. Three blocker oligonucleotides (29 to 31 bases) hybridize to the complementary strand (see Suppl. Info. for details). All DNA sequences used in this study are listed in Table 1. After 2 min at 95°C to ensure the denaturation of the double-stranded DNA target and to prevent potential hairpins or autohybridized DNA, the hybridization to capture and detection probes and to blockers was achieved in a thermocycler by lowering the temperature from 55°C to 25°C at a rate of 1°C per min.

#### Immobilization

10 µl of activated streptavidin coated magnetic micro-beads were added to the hybridization solution for immobilization under gentle continuous stirring for 30 min at room temperature (see Suppl. Info. for details). The beads were washed twice in 150 µl of a 0.1xSSC, Tween 20 (0.5%) buffer using a magnet to remove the unbound material. After resuspension in 60 µl of the same buffer, microbeads were finally heated at 95°C for 20 min for denaturation of both the DNA hybridized triplex and the biotin-streptavidin bound [37]. The microbeads were then immobilized on the magnet and the supernatant containing the SERRS probe was collected for SERRS measurements.

#### SERRS measurements

Surface-Enhanced Resonant Raman Spectroscopy relies on the amplification of the Raman signal of a compound of interest up to a factor of 10^14^ [38], [39]. SERRS-active compounds adsorb onto a rough metallic substrate, which quenches the molecule's fluorescence, and amplifies its Raman signal. Further amplification is obtained with an excitation wavelength close to the maximum absorption frequency of the molecule due to a resonance effect [31]. Here, the labeled detection probe adsorbs onto the nanoparticles of a silver colloid, synthesized following the Lee and Meisel protocol [40] (see Suppl. Info. for details). The silver colloid has been stored in the dark at room temperature. All silver colloid aliquots used in this study are from the same batch. The eluted R6G SERRS probes contained in the supernatant after the assay were analyzed following Feuillie *et al.* [34]. 20 µl of the supernatant was mixed with 20 µl of spermine (10^−2^ mol.l^−1^) in a single-use PMMA spectroscopy cuvette. Spermine acts as an aggregating agent for the silver nanoparticles, thus creating hot spots of amplification, and also neutralizes the negative charges of the DNA backbone for a better adsorption onto the silver nanoparticles [31]. 500 µl of silver colloid and 500 µl of distilled water were added and the solution was then homogenized prior to SERRS measurement. Samples were analyzed with a Horiba Jobin Yvon LabRam HR 800 Raman spectrometer, coupled to a Spectra Physics 2018 Ar^+^/Kr^+^ 24 laser tuned at 514.5 nm (LGL, ENS de Lyon). The excitation wavelength is therefore close to the maximum absorption wavelength of R6G, 524 nm [41]. The laser power on the sample was adjusted between 1.5 and 2 mW. Spectra were acquired in 30 s with a spectrometer grating of 600 gr.mm^−1^ centered at 1600 cm^−1^.

#### Quantification of the amount of DNA target

SERRS spectra are processed with the Peakfit software. R6G is easily identified thanks to a series of intense Raman peaks [34], [42], [43]. The parameter chosen for quantification is the area of the most intense peak centered at 1650 cm^−1^, noted *A_1650_*. The SERRS-hybridization assay allows quantifying the amount of probe in the elution solution, as previously showed [36]. With a non-degraded target DNA, this parameter has a positive correlation with the amount of target in the initial solution.

### PCR assay

A standard PCR amplification was performed using the hot-start *AmpliTaq Gold polymerase* (Lifetechnologies, # N8080245) often used in ancient DNA studies. This enzyme leads to higher amplification yields than standard *Taq* Polymerase [44]. PCR primers of 21 and 18 bases, Rup_For_5′_ and Rup_Rev_3′_ respectively, were designed to amplify specifically a region of 134 bp of our target DNA, (mitochondrial 12S rRNA gene of *Rupicapra rupicapra* (Chamois)). Primers are listed in Table 1. PCR mixes were prepared in a clean room dedicated to ancient DNA studies (Palgene platform, Lyon) to avoid external contaminations. For each double-stranded DNA molecule studied, each representing a different level of DNA degradation, four independent PCR reactions were carried out. Each PCR reaction consists of a 10 min activation step at 94°C followed by 45 cycles of denaturation at 95°C for 30 s, annealing at 58°C for 30 s, and extension at 72°C for 30 s, and a final extension step at 72°C for 7 min. PCR were also conducted without DNA template as a control to monitor DNA contaminations and/or primer dimers formation. PCR products were first separated on 2% high-resolution agarose gel electrophoresis stained with ethidium bromide and then visualised under UV illumination. Positive PCR products were subsequently cloned using the Topo TA cloning kit (Invitrogen) following the manufacturer instructions and sequenced by Beckman Genomics (see Suppl. Info. for details). Sequences were aligned manually using the Seaview software [45].

## Results and Discussion

### PCR amplification of altered DNA

#### Characteristics of the PCR products

**Figure 2:**
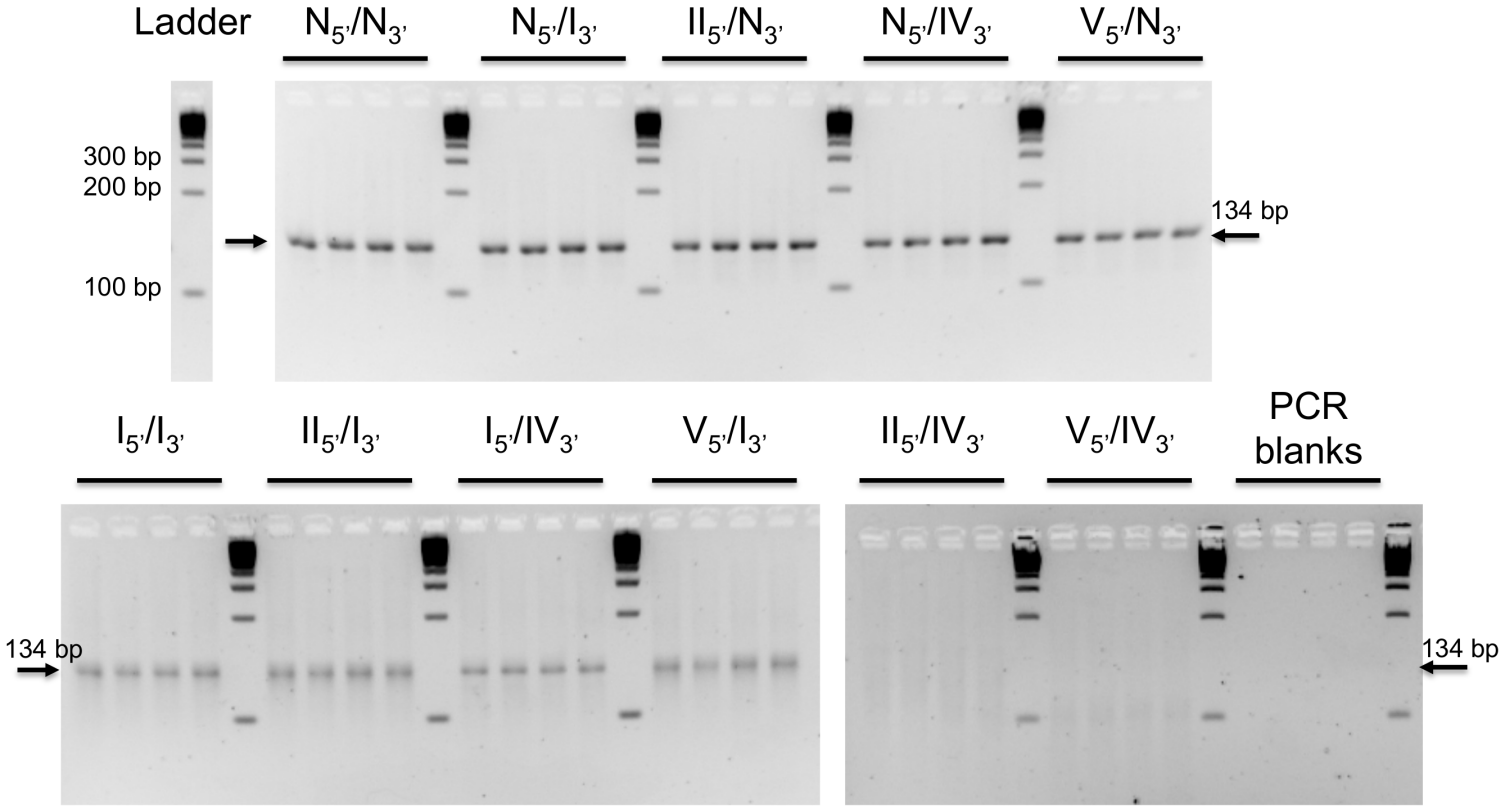
High-resolution agarose gel electrophoresis of PCR products obtained from amplification of eleven double-stranded DNA molecules containing abasic sites. PCR conditions are given in the “*Material and Methods: PCR conditions*” part. Four independent PCR reactions were carried out for each degraded DNA template using Rup_For_5′_ and Rup_Rev_3′_ primers (expected fragment size  =  134 bp). The size of some DNA markers in bp is indicated. All gels were revealed using the same transilluminator settings.

**Figure 3:**
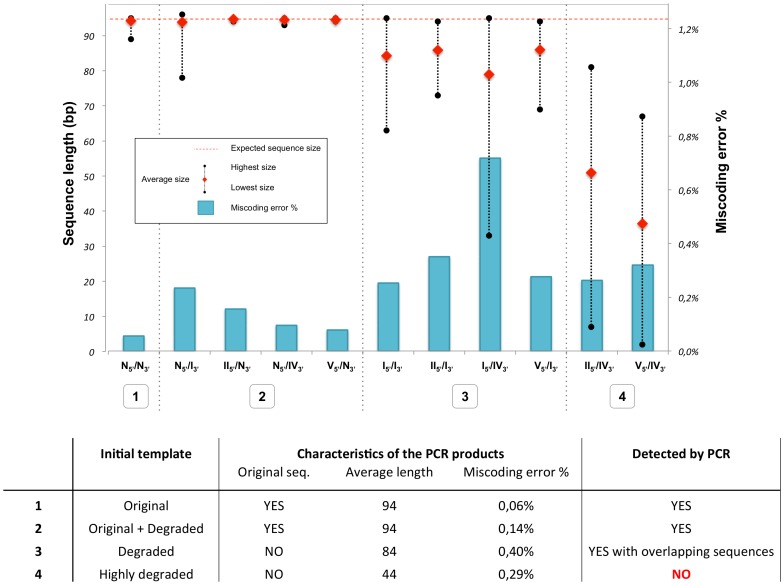
Errors of amplification observed in the PCR products. Two types of errors were found: deletions and miscoding errors. The weigthed average length of the PCR products is represented in red diamonds. The dotted bar represent the dispersion of obtained length. Primer sequences have been deleted from the analysis. Therefore, the full length expected sequence, figured by a red dashed line, is here 95 bp instead of 134 bp. The blue histogram represents the percentage of miscoding errors retrieved in PCR products.

The eleven double-stranded molecules (Table 2) were used as templates for PCR amplification. Primers were designed to amplify a fragment of 134 bp of mitochondrial DNA of *Rupicapra rupicapra* (12S rRNA gene) corresponding to the target DNA molecule of our study. PCR products were characterized both by high-resolution gel electrophoresis, presented in Figure 2, and by sequencing. Alignments of the sequences obtained for the eleven double-stranded molecules are available in the Supplementary Information (Tables S1–S11, in File S1), and have allowed us to detect errors and deletions in the PCR products. These results are presented in Figure 3. Since primers have been introduced in the system, their sequence was removed from analysis. The expected length considered here is therefore 95, *i.e.* the target length 134 bp, minus both primers of 21 and 18 bases, respectively. The miscoding error percentage is the ratio of misincorporated nucleotides in the final PCR products over the theoretical total number of bases in case of perfect amplification.

Contamination and formation of primer dimers were ruled out by PCR controls (Figure 2). Electrophoresis gels reveal a single specific and intense band of the expected size 134 bp for PCR results of molecules with at least one native non-degraded N_5′_ or N_3′_ strand (Figure 2). Indeed, sequencing results show that perfect copies of the original molecules are found in majority in the case of the amplification of the non-degraded N_5′_/N_3′_ molecule, as well as for the amplification of molecules with one non-degraded strand (*i.e.* N_5′_/I_3′_, II_5′_/N_3′_, N_5′_/IV_3′_ and V_5′_/N_3′_, see Tables S2, S3, S4 and S5 respectively, in File S1). When one strand is degraded, the miscoding error percentage is however in average 2.3 times higher than for the original N_5′_/N_3′_ molecule, and short scale deletions (1 to 17 bases) are more frequent (Figure 3).

PCR products of molecules with 2 degraded strands are characterized by a further increase of miscoding errors, combined with a decrease of the average length of the sequences (Figure 3). The sequencing results reveal that the original molecule is no longer present in the amplicons (Tables S6–S11, in File S1). The miscoding error percentage reaches an average of 0.36%, which is more than twice higher than for molecules with one native N strand (Figure 3). These miscoding errors cannot be ascribed to the intrinsic error rate of 2.6×10^−5^ of the *AmpliTaq Gold* DNA polymerase only [46] and therefore originate from the altered parts of the DNA template. The analysis of the deletion content of all molecules with two degraded strands allows distinguishing between two groups of molecules. First, the molecules I_5′_/I_3′_, II_5′_/I_3′_, I_5′_/IV_3′_ and V_5′_/I_3′_ lead to PCR products of a mean size close to the expected one and display a slight smear (diffuse electrophoresis band, due to the presence of DNA molecules of diverse low sizes) (Figure 2). The sequences obtained are on average 10 bases shorter than the expected sequence (Figure 3). Second, the most degraded molecules II_5′_/IV_3′_ and V_5′_/IV_3′_ reveal no specific PCR amplification at or close to the expected size. They exhibit only a broad smear (Figure 2) and the corresponding sequences display large-scale deletions (Tables S10 and S11, in File S1). The lengths of the sequences are in average 45 and 59 bases shorter than expected for the II_5′_/IV_3′_ and V_5′_/IV_3′_ molecules, respectively (Figure 3). Practically, these two most degraded molecules cannot be specifically detected by PCR amplification and the specific targeted sequence cannot be deduced from overlapping PCR products, as they've undergone too many and too large deletions (Tables S10 and S11, in File S1).

#### PCR biases

The deleted areas in the PCR products are clearly located at or nearby the original abasic sites locations (*e.g.* Tables S6 and S11, in File S1). Sequencing results allow us to highlight two major PCR biases. First, the PCR primer Rup_For_5′_ is obviously favored, most probably during PCR annealing. It results in a better amplification of the R_3′_ strand compared to the R_5′_ strand, as shown by the PCR and sequencing results of the I_5′_/I_3′_ molecule (Table S6, in File S1). The original I_5′_/I_3′_ molecule displays one abasic site at position 74 on the I_5′_ strand, and one abasic site at position 48 on the I_3′_ strand (Table 1). A large majority of PCR products of this template exhibit deletions or miscoding errors around the 48^th^ position (19/25), whereas only 4/25 sequences bear modifications around the 74^th^ position (Table S6 in File S1). This clearly shows that the amplification of the 3′-5′ strand I_3′_ is favored. More generally, the amplification of the R_3′_ strand is systematically preferred in the molecules carrying both degraded strands (Tables S2–S11 in File S1). Second, we also found evidence of jumping PCR, illustrated by PCR event n°9 of the I_5′_/I_3′_ template, where the sequences exhibit deletions around both the 48^th^ and 74^th^ positions (Table S6 in File S1). A chimeric molecule has been created using more than one template. Here, both strands of the initial DNA molecule were used and the amplification product therefore bears both modifications.

To summarize, theses results show that even a sample rich in DNA may not lead to a successful PCR amplification due to the alteration of the DNA template. The failure of PCR on such a simple synthetic model substrate emphasizes the need for an alternate non-enzymatic DNA detection method, such as the SERRS-hybridization assay proposed in this study.

### Detection of altered DNA by the SERRS-hybridization assay

**Figure 4:**
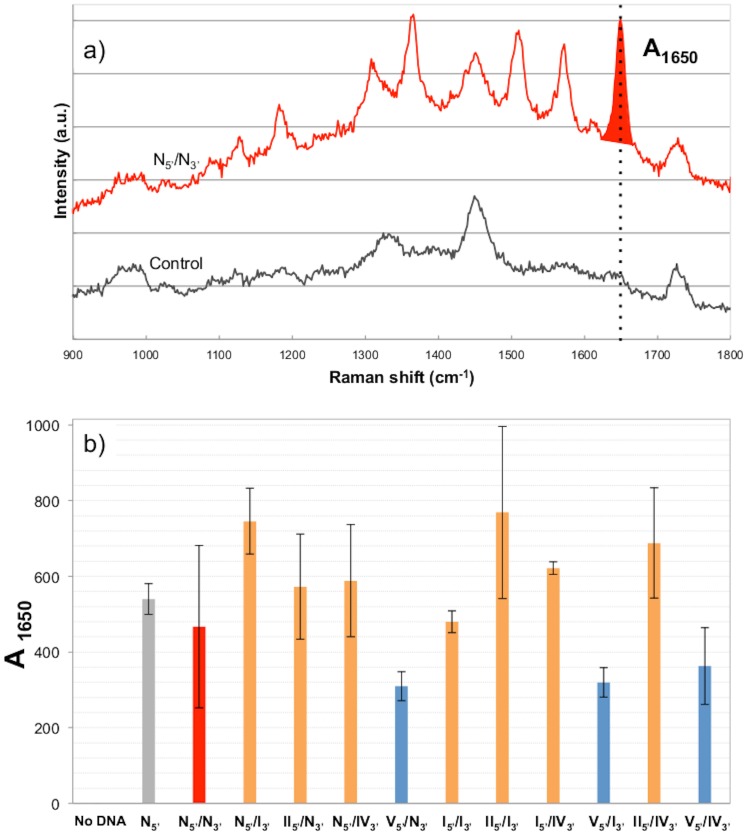
SERRS-hybridization detection results. a) SERRS spectra obtained for the analysis of the non-degraded double stranded molecule N_5′_/N_3′_ (in red), and a control solution void of target DNA (in black). The parameter used for quantification is the area of the most intense Raman band of Rhodamine 6G at 1650 cm^−1^, and is noted A_1650_. Target: 5×10^−8^M; - Blockers: 5×10^−5^M; - Capture and detection probe: 10 mM. The peaks visible on the control spectrum come from the PMMA cuvettes used for the measurements. b) SERRS-hybridization assay detection results. In grey is the SERRS signal obtained for the single-stranded non-degraded DNA sequence N_5′_. In red is the SERRS signal obtained for the detection of the non-degraded double-stranded N/N molecule. Degraded molecules appear in orange, except for molecules containing the V_5′_ strand with 5 abasic sites, that appear in blue. All degraded molecules are detected with signals comparable to those of non-degraded molecules. Concentrations used in this study: - Target: 5×10^−8^M; - Blockers: 5×10^−5^M; - Capture and detection probe: 10 mM. Error bars are 2 standard deviation.

The same eleven molecules with an alteration degree ranging from no abasic sites in the original sequence to 9 abasic sites distributed on both strands in the most degraded molecule were investigated by the SERRS-hybridization assay. The specificity of the detection/capture system has been previously tested [34] and allows the specific detection of the target DNA sequence of *Rupicapra rupicapra*, even in a mixture with an analog sequence of mitochondrial DNA (12S RNA gene) of a closely related species, the common goat (*Capra hircus*). The signal of the detection probe, labeled with R6G, is observed only when target DNA is present in the analyzed sample (Figure 4 a). The amount of target DNA detected is quantified by the area of the most intense Raman band of Rhodamine 6G, centered at 1650 cm^−1^ and hereafter noted A_1650_ (Figure 4 a).

Figure 4 b presents the detection levels achieved for the same concentration of degraded templates previously characterized by PCR. The SERRS spectra obtained for negative controls void of DNA do not display any signal characteristic of R6G, thus ruling out adsorption or non-specific hybridization. We also verified that the system of three blockers was completely hindering the rehybridization between complementary strands by comparing the SERRS results obtained for double-stranded and for the single-stranded original sequence. The SERRS signals obtained at the same concentration have similar intensities (Figure 4 b), which indicates that the blocking architecture is highly efficient.

The analyses of the degraded molecules by the SERRS-hybridization assay all lead to a strong SERRS signal from the R6G probe, with amplitudes comparable to the signal obtained for the original non-degraded N_5′_/N_3′_ molecule (Figure 4 b). Regardless of their respective degradations, all ten degraded molecules are detected by the SERRS-hybridization assay without any doubt, even the most degraded ones II_5′_/IV_3′_ and V_5′_/IV_3′_ that PCR failed to amplify (Figure 2, Figure 3). The present SERRS measurements therefore show that the SERRS-hybridization assay detects the presence of a specific target DNA sequence, even when degraded and refractory to PCR.

Two aspects of the robustness of the SERRS-hybridization assay for the detection of degraded DNA were tested. First, the blocking system was challenged by locating abasic sites in the hybridization region of one of the blockers, Block2. Indeed, the IV_3′_ strand presents three abasic sites towards the middle blocker Block2 (Table 1). The hybridization of this blocker to the target DNA is therefore compromised. When analyzed by the SERRS-hybridization assay, the three DNA molecules N_5′_/IV_3′_, I_5′_/IV_3′_, II_5′_/IV_3′_ are nevertheless detected without any alteration of the SERRS signal compared to the N_5′_/N_3′_ molecule, despite the abasic sites located in the hybridization area of the blocker Block2 (Figure 4b). The system of three blockers allows a full access to the target strand, even when the hybridization of one of them may not be guaranteed. Second, we challenged the capture system by placing abasic sites in the capture probe hybridization zone. Although the most degraded target strand V_5′_ has two abasic sites in the capture probe hybridization area (Table 1), we still observe an intense SERRS signal for the templates containing this strand, *i. e.* V_5′_/N_3′_, V_5′_/I_3′_ and V_5′_/IV_3′_. However, these 3 molecules systematically present a 23 to 34% weaker SERRS signal than the original molecule. When abasic sites are located in the capture zone, the immobilization of target molecules thus appears less efficient. Some target DNA might be left “uncaught” in the sample, or lost during the washing step, as the specificity of the capture probe hybridization could be slightly decreased. Nevertheless the obtained SERRS signal clearly indicates the presence of the targeted molecules in the sample despite their high level of degradation and the decreased efficiency of the capture process. The SERRS-hybridization assay is therefore a powerful non-enzymatic tool that could be applied as a first test prior to any enzymatic amplification or reparation procedure to assess the presence of target DNA in a degraded/ancient or processed sample. To the best of our knowledge, this is the first time that degraded DNA that was not suitable for routine analysis by PCR was directly detected without DNA repair. This biophysical approach therefore provides an alternative to enzyme-based DNA detection tools such as PCR. DNA extracts from a potentially degraded sample could be tested without further treatment (purification, amplification, reparation…) by the SERRS-hybridization assay. Moreover, thanks to its multiplexing possibilities [36], several target DNA could be tested at the same time, in the case of unidentified bone fragments for instance, thus saving time and money.

## Conclusion

In the present study, we have succeeded in detecting altered DNA molecules that PCR failed to detect. This is the first time that DNA refractory to PCR can be detected prior to any repair process. Through a fully non-enzymatic method, based on molecular hybridization and SERRS detection, we achieved the detection of a range of double-stranded DNA molecules containing between 0 and 9 abasic sites distributed on both strands. Although the target DNA concentration was high, the *AmpliTaq Gold* polymerase could not correctly bypass the lesions, leading either to small-scale deletions and miscoding incorporations, or to a complete failure of amplification for the most degraded molecules. The analysis of the same molecules by the SERRS-hybridization assay lead to the detection of the whole range of degraded molecules with SERRS signal intensities comparable to those observed for the authentic non-degraded molecules. We have therefore proven that this SERRS-hybridization assay has the potential to analyze degraded samples and therefore to enlarge the number and variety of extracts suitable for further DNA analyses

In the future, samples could be quickly tested for the presence of a specific double-stranded target DNA by the SERRS-hybridization assay before getting involved in long and expensive trials of DNA purification and repair. This could be particularly useful in the case of ancient DNA, where DNA is often degraded and co-extracted with polymerase inhibitors, and where there is no way to assess the amount of DNA present in a sample prior to enzymatic procedures. Moreover, given the multiplexing capacity of the SERRS-hybridization assay, multiple DNA targets could be screened in one trial [36] even when highly degraded. The biophysical approach used in the SERRS-hybridization assay enables access to a new range of samples that were not suitable for PCR analysis because of high degradation. Furthermore, though degradation content is not directly correlated to the age of a DNA sample, the SERRS-hybridization assay could help in the search for older DNA biosignatures. Finally, this assay could also have many applications in research areas such as medical diagnosis. Specific mRNA relative to cancers could be targeted and detected by the SERRS-hybridization assay in blood samples without purification, despite a high amount of PCR inhibitors [47].

## Supporting Information

File S1 **Tables S1–S11.** **Table S1. Alignment of the sequences obtained from cloning-sequencing for the PCR amplification of the N_5′_/N_3′_ molecule.** The N molecule is taken as a reference. PCR amplification primers are removed from the analysis and appear only on the template molecule in red. Differences from the initial molecule are represented in red. For each haplotype sequence, the observed frequency and percentage are given. **Table S2. Alignment of the sequences obtained from cloning-sequencing for the PCR amplification of the N_5′_/I_3′_ molecule.** The N_5′_ and C_I_3′_ molecules are taken as a reference. The C_I_3′_ corresponds to I_3′_ strand in the reverse complementary orientation. **Table S3. Alignment of the sequences obtained from cloning-sequencing for the PCR amplification of the II_5′_/N_3′_ molecule.** The II_5′_ and C_N_3′_ molecules are taken as a reference. The C_N_3′_ corresponds to N_3′_ strand in the reverse complementary orientation. **Table S4. Alignment of the sequences obtained from cloning-sequencing for the PCR amplification of the N_5′_/IV_3′_ molecule.** The N_5′_ and C_IV_3′_ molecules are taken as a reference. The C_IV_3′_ corresponds to IV_3′_ strand in the reverse complementary orientation. **Table S5. Alignment of the sequences obtained from cloning-sequencing for the PCR amplification of the V_5′_/N_3′_ molecule.** The V_5′_ and C_N_3′_ molecules are taken as a reference. The C_N_3′_ corresponds to N_3′_ strand in the reverse complementary orientation. **Table S6. Alignment of the sequences obtained from cloning-sequencing for the PCR amplification of the I_5′_/I_3′_ molecule.** The I_5′_ and C_I_3′_ molecules are taken as a reference. The C_I_3′_ corresponds to I_3′_ strand in the reverse complementary orientation. **Table S7. Alignment of the sequences obtained from cloning-sequencing for the PCR amplification of the II_5′_/I_3′_ molecule.** The II_5′_ and C_I_3′_ molecules are taken as a reference. The C_I_3′_ corresponds to I_3′_ strand in the reverse complementary orientation. **Table S8. Alignment of the sequences obtained from cloning-sequencing for the PCR amplification of the I_5′_/IV_3′_ molecule.** The I_5′_ and C_IV_3′_ molecules are taken as a reference. The C_IV_3′_ corresponds to IV_3′_ strand in the reverse complementary orientation. **Table S9. Alignment of the sequences obtained from cloning-sequencing for the PCR amplification of the V_5′_/I_3′_ molecule.** The V_5′_ and C_I_3′_ molecules are taken as a reference. The C_I_3′_ corresponds to I_3′_ strand in the reverse complementary orientation. **Table S10. Alignment of the sequences obtained from cloning-sequencing for the PCR amplification of the II59/IV39 molecule.** The II59 and C_IV39 molecules are taken as a reference. The C_IV39 corresponds to IV39 strand in the reverse complementary orientation. **Table S11. Alignment of the sequences obtained from cloning-sequencing for the PCR amplification of the V59/IV39 molecule.** The V59 and C_IV39 molecules are taken as a reference. The C_IV39 corresponds to IV39 strand in the reverse complementary orientation.
(DOCX)

## References

[1] Wolf C, Rentsch J, Hübner P (1999) PCR-RFLP Analysis of Mitochondrial DNA: A Reliable Method for Species Identification. J Agric Food Chem 47:1350–1355. 10563979 10.1021/jf9808426

[2] Fajardo V, Gonzalez I, Lopez-Calleja I, Martin I, Hernandez P, et al (2006) PCR-RFLP Authentication of Meats from Red Deer (Cervus elaphus), Fallow Deer (Dama dama), Roe Deer (Capreolus capreolus), Cattle (Bos taurus), Sheep (Ovis aries), and Goat (Capra hircus). J Agric Food Che 54:1144–1150. 10.1021/jf051766r 16478229

[3] Wozney KM, Wilson PJ (2012) Real-time PCR detection and quantification of elephantid DNA: Species identification for highly processed samples associated with the ivory trade. Forensic Science International 219:106–112. 22257967 10.1016/j.forsciint.2011.12.006

[4] Alaeddini R, Walsh SJ, Abbas A (2010) Forensic implications of genetic analyses from degraded DNA–A review. Forensic Science International: Genetics 4:148–157. 20215026 10.1016/j.fsigen.2009.09.007

[5] Newman ME, Parboosingh JS, Bridge PJ, Ceri H (2002) Identification of Archaeological Animal Bone by PCR/DNA Analysis. Journal of Archaeological Science 29:77–84.

[6] Barnes I, Young JPW, Dobney KM (2000) DNA-based Identification of Goose Species from Two Archaeological Sites in Lincolnshire. Journal of Archaeological Science 27:91–100.

[7] Höss M, Dilling A, Currant A, Pääbo S (1996) Molecular phylogeny of the extinct ground sloth *Mylodon darwinii*. Proc Natl Acad Sci USA 93, pp.181–185. 10.1073/pnas.93.1.181 PMC40202 8552600

[8] Pruvost M, Schwarz R, Bessa Correia V, Champlot S, Braguier S, et al (2007) Freshly excavated fossil bones are best for amplification of ancient DNA. PNAS 104 no. 3:739–744. 17210911 10.1073/pnas.0610257104 PMC1783384

[9] Heyn P, Stenzel U, Briggs AW, Kircher M, Hofreiter M, et al (2010) Road blocks on paleogenomes-polymerase extension profiling reveals the frequency of blocking lesions in ancient DNA. Nucleic Acids Research 38 no. 16:e161. 20587499 10.1093/nar/gkq572 PMC2938203

[10] Pääbo S (1989) Ancient DNA - Extraction, Characterization, Molecular-Cloning, and Enzymatic Amplification. P Natl Acad Sci USA 86:1939–1943. 10.1073/pnas.86.6.1939 PMC286820 2928314

[11] Pääbo S, Poinar H, Serre D, Jaenicke-Despres V, Hebler J, et al (2004) Genetic analyses from ancient DNA, Annu. Rev Genet 38:645–79. 10.1146/annurev.genet.37.110801.143214 15568989

[12] Lindahl T, Andersson A (1972) Rate of chain breakage at apurinic sites in double-stranded deoxyribonucleic acid. Biochemistry. vol 11, no. 19:3618–3623. 10.1021/bi00769a019 4559796

[13] Höss M, Jaruga P, Zastawny TH, Dizdaroglu M, Pääbo S (1996) DNA damage and DNA sequence retrieval from ancient tissues. Nucleic Acids Research 24:1304–1307. 8614634 10.1093/nar/24.7.1304 PMC145783

[14] Willerslev E, Cooper A (2005) Ancient DNA. P Roy Soc B-Biol Sci 272:3–16. 10.1098/rspb.2004.2813 PMC1634942 15875564

[15] Sikorsky JA, Primerano DA, Fenger TW, Denvir J (2004) Effect of DNA damage on PCR amplification efficiency with the relative threshold cycle method. Biochem Bioph Res Co 323:823–830. 10.1016/j.bbrc.2004.08.168 15381074

[16] Sikorsky JA, Primerano DA, Fenger TW, Denvir J (2007) DNA damage reduces Taq DNA polymerase fidelity and PCR amplification efficiency. Biochem Bioph Res Co 355:431–437. 10.1016/j.bbrc.2007.01.169 PMC1945218 17303074

[17] Haracska L, Washington MT, Prakash S, Prakash L (2001) Inefficient bypass of an abasic site by DNA polymeraseη eta. J Biol Chem 276:6861–6866. 11106652 10.1074/jbc.M008021200

[18] Pääbo S, Irwin DM, Wilson AC (1990) DNA damage promotes jumping between templates during enzymatic amplification. The journal of biological chemistry vol 265: no. 8, pp. 4718–4721. 2307682

[19] Brotherton P, Endicott P, Sanchez JJ, Beaumont M, Barnett R, et al. (2007) Novel high-resolution characterization of ancient DNA reveals C′U-type base modification events as the sole cause of post mortem miscoding lesions. Nucleic Acids Research Vol. 35: No. 17, 5717–5728. 10.1093/nar/gkm588 PMC2034480 17715147

[20] Shibutani S, Takeshita M, Grollman AP (1997) Translesional synthesis on DNA templates containing a single abasic site - A mechanistic study of the ''A rule''. J Biol Chem 272:13916–13922. 9153253 10.1074/jbc.272.21.13916

[21] Brotherton P, Endicott P, Beaumont M, Barnett R, Austin J, et al (2008) Single primer extension (SPEX) amplification to accurately genotype highly damaged DNA templates. Forensic Sci. Int.: Genetics suppl. Series 1:19–21.

[22] Rizzi E, Lari M, Gigli E, De Bellis G, Caramelli D (2012) Ancient DNA studies: new perspectives on old samples. Genetics Selection Evolution 44:21. 10.1186/1297-9686-44-21 PMC3390907 22697611

[23] Briggs AW, Stenzel U, Meyer M, Krause J, Kircher M, et al (2010) Removal of deaminated cytosines and detection of in vivo methylation in ancient DNA. Nucleic acid research vol 38, no. 6:e87. 10.1093/nar/gkp1163 PMC2847228 20028723

[24] D′Abbadie M, Hofreiter M, Vaisman A, Loakes D, Gasparutto D, et al. (2007) Molecular breeding of polymerases for amplification of ancient DNA. Nature biotech 25, pp.939–943. 10.1038/nbt1321 PMC1978225 17632524

[25] McDonald JP, Hall A, Gasparutto D, Cadet J, Ballantyne J, et al. (2006) Novel thermostable Y-family polymerases: applications for the PCR amplification of damaged or ancient DNAs. Nucleic acids research vol 34 no. 4. 10.1093/nar/gkj512 PMC1373694 16488882

[26] Gloeckner C, Sauter K BM, Marx A (2007) Evolving a thermostable DNA polymerase that amplifies from highly damaged templates, Angew Chem Int Ed. 46:3115–3117. 10.1002/anie.200603987 17366498

[27] Zhao XJ, Tapec-Dytioco R, Tan WH (2003) Ultrasensitive DNA detection using highly fluorescent bioconjugated nanoparticles. J Am Chem Soc 125:11474–11475. 13129331 10.1021/ja0358854

[28] Song C, Yang X, Wang K, Wang Q, Huang J, et al (2014) Label-free and non-enzymatic detection of DNA based on hybridixation chain reaction amplification and dsDNA-templated copper nanoparticles. Analytica Chimica Acta 827:74–79. 24832997 10.1016/j.aca.2014.04.006

[29] Taton TA, Mirkin CA, Letsinger RL (2000) Scanometric DNA array detection with nanoparticle probes. Science 289:1757–1760. 10976070 10.1126/science.289.5485.1757

[30] Storhoff JJ, Marla SS, Bao P, Hagenow S, Mehta H, et al (2004) Gold nanoparticle-based detection of genomic DNA targets on microarrays using a novel optical detection system. Biosens Bioelectron 19:875–883. 15128107 10.1016/j.bios.2003.08.014

[31] Graham D, Smith WE, Linacre AMT, Munro CH, Watson ND, et al (1997) Selective detection of deoxyribonucleic acid at ultralow concentrations by SERRS. Anal Chem 69:4703–4707.

[32] Faulds K, McKenzie F, Smith WE, Graham D (2007) Quantitative simultaneous multianalyte detection of DNA by dual-wavelength surface-enhanced resonance Raman scattering. Angew Chem Int Edit 46:1829–1831. 10.1002/anie.200604265 17262874

[33] Monaghan PB, Graham D, McCarney KM, Ricketts A, Littleford RE, et al (2007) Bead-based DNA diagnostic assay for chlamydia using nanoparticle-mediated surface-enhanced resonance Raman scattering detection within a lab-on-a-chip format. Anal Chem 79:2844–2849. 17326610 10.1021/ac061769i

[34] Feuillie C, Merheb MM, Gillet B, Montagnac G, Daniel I, et al (2011) A novel SERRS sandwich-hybridization assay to detect specific DNA target. Plos One 6:e17847. 21655320 10.1371/journal.pone.0017847 PMC3104981

[35] Hill HD, Vega RA, Mirkin CA (2007) Nonenzymatic detection of bacterial genomic DNA using the Bio Bar Code assay. Anal Chem 79:9218–9223. 17927207 10.1021/ac701626y PMC3241528

[36] Feuillie C, Merheb MM, Gillet B, Montagnac G, Hanni C, et al (2012) Enzyme-free detection and quantification of double-stranded nucleic acids. Anal Bioanal Chem 10.1007/s00216-012-6133-1 22695500

[37] Holmberg A, Blomstergren A, Nord O, Lukacs M, Lundeberg J, et al (2005) The biotin-streptavidin interaction can be reversibly broken using water at elevated temperatures. Electrophoresis 26:501–510. 15690449 10.1002/elps.200410070

[38] Nie S, Emory SR (1997) Probing single molecules and single nanoparticles by Surface-Enhanced Raman Scattering. Science 275:1102. 9027306 10.1126/science.275.5303.1102

[39] Maher RC, Cohen LF, Etchegoin P (2002) Single molecule photo-bleaching observed by surface enhanced resonant Raman scattering (SERRS). Chemical physics letters 352:378–384.

[40] Lee PC, Meisel D (1982) Adsorption and Surface-Enhanced Raman of Dyes on Silver and Gold Sols. J Phys Chem-Us 86:3391–3395.

[41] Graham D, Faulds K (2008) Quantitative SERRS for DNA sequence analysis. Chem Soc Rev 37:1042–1051. 18443688 10.1039/b707941a

[42] Faulds K, Graham D, Smith WE (2004) Evaluation of surface-enhanced resonance Raman scattering for quantitative DNA analysis. Anal Chem 76:412–417. 14719891 10.1021/ac035060c

[43] Jensen L, Schatz GC (2006) Resonance Raman scattering of rhodamine 6G as calculated using time-dependent density functional theory. J Phys Chem A 110:5973–5977. 16671663 10.1021/jp0610867

[44] Moretti T, Koons B, Budowle B (1998) Enhancement of PCR amplification yield and specificity using AmpliTaq Gold™ DNA polymerase. Biotechniques 25:716–722. 9793657

[45] Gouy M, Guindon S, Gascuel O (2010) SeaView Version 4: A Multiplatform Graphical User Interface for Sequence Alignment and Phylogenetic Tree Building. Mol Biol Evol 27:221–224. 19854763 10.1093/molbev/msp259

[46] Beaulieu M, Larson GP, Geller L, Flanagan SD, Krontiris TG (2001) PCR candidate region mismatch scanning: adaptation to quantitative, high-throughput genotyping. Nucleic Acids Research 29:1114–1124. 11222761 10.1093/nar/29.5.1114 PMC29718

[47] Radström P, Knutsson R, Wolffs P, Lövenklev M, Löfström C (2004) Pre-PCR processing, Strategies to generate PCR-compatible samples. Molecular biotechnology 26:133–146. 14764939 10.1385/MB:26:2:133

