# Supplementary material for: Detection of DNA Sequences Refractory to PCR Amplification Using a Biophysical SERRS Assay (Surface Enhanced Resonant Raman Spectroscopy)
Source: PLoS One. 2014 Dec 12;9(12):e114148. doi: 10.1371/journal.pone.0114148 (PMC4264738; doi:10.1371/journal.pone.0114148)
Supplement: File S1 — Tables S1–S11. Table S1. Alignment of the sequences obtained from cloning-sequencing for the PCR amplification of the N5′/N3′ molecule. The N molecule is taken as a reference. PCR amplification primers are removed from the analysis and appear only on the template molecule in red. Differences from the initial molecule are represented in red. For each haplotype sequence, the observed frequency and percentage are given. Table S2. Alignment of the sequences obtained from cloning-sequencing for the PCR amplification of the N5′/I3′ molecule. The N5′ and C_I3′ molecules are taken as a reference. The C_I3′ corresponds to I3′ strand in the reverse complementary orientation. Table S3. Alignment of the sequences obtained from cloning-sequencing for the PCR amplification of the II5′/N3′ molecule. The II5′ and C_N3′ molecules are taken as a reference. The C_N3′ corresponds to N3′ strand in the reverse complementary orientation. Table S4. Alignment of the sequences obtained from cloning-sequencing for the PCR amplification of the N5′/IV3′ molecule. The N5′ and C_IV3′ molecules are taken as a reference. The C_IV3′ corresponds to IV3′ strand in the reverse complementary orientation. Table S5. Alignment of the sequences obtained from cloning-sequencing for the PCR amplification of the V5′/N3′ molecule. The V5′ and C_N3′ molecules are taken as a reference. The C_N3′ corresponds to N3′ strand in the reverse complementary orientation. Table S6. Alignment of the sequences obtained from cloning-sequencing for the PCR amplification of the I5′/I3′ molecule. The I5′ and C_I3′ molecules are taken as a reference. The C_I3′ corresponds to I3′ strand in the reverse complementary orientation. Table S7. Alignment of the sequences obtained from cloning-sequencing for the PCR amplification of the II5′/I3′ molecule. The II5′ and C_I3′ molecules are taken as a reference. The C_I3′ corresponds to I3′ strand in the reverse complementary orientation. Table S8. Alignment of the sequences obtained from cl [file pone.0114148.s001.docx]

**Supporting Information**

**Experimental precisions**

**PCR assay**

*Primer design*. PCR primers of 21 and 18 bases, Rup_For_5’_ and Rup_Rev_3’_ respectively, were designed to amplify specifically a fragment of 134 bases of mitochondrial 12S rRNA gene of *Rupicapra rupicapra* (Chamois). Primers are listed in Table 1 and were purchased from Eurogentec^®^.

*PCR conditions.* PCR reaction was carried out in a total volume of 25 μl. For one reaction, PCR conditions were as follows: 0.5 μl of Taq Gold polymerase (5U/μl, Applied-Biosystems^®^), 2.5 μl of 10X Gold buffer, 2.5 μl of MgCl_2_ (25 mM), 0.5 μl of each primer Rup_For_5’_ and Rup_Rev_3’_ (25 μM each), 0.25 μl of dNTP (100 μM) and 1.5 µl of DNA target (5x10^-8^ M). The following thermal cycling program was conducted: an initial activation of hot-start DNA Polymerase at 95°C for 5 min, followed by 45 cycles of denaturation at 95°C for 30 s, annealing at 58°C for 30 s, and extension at 72°C for 30 s, followed by a final extension step at 72°C for 7 min. Five µl of PCR products were visualised on high-resolution agarose gels (2% agarose / 4% NuSieve^®^ GTG^®^ agarose) using ethidium bromide staining and UV light transillumination.

*Cloning and sequencing.* PCR products were cloned into bacterial vectors using TOPO TA cloning for sequencing kit^®^ (Life Technologies™, #K457540) according to the manufacturers instructions. Screen for plasmid inserts was directly conducted from *E. coli* colonies using PCR amplification. PCR reaction was carried out in a total volume of 20 μl. For one reaction, PCR conditions were as follows: 8 μl of Master Mix (5 Prime), 0.25 μl of each M13 Rev1 and M13 For1 primers (10 μM each) and 11.5 μl of sterile water. M13 Rev1 (5’- GTTTTCCCAGTCACGACGTTG -3’) and M13 For1 (5’- TTTCACACAGGAAACAGCTAT -3’) primers flank the cloning site. For each cloned amplicon, 8 bacterial colonies were directly tooth-picked into PCR reaction mix and were screened by PCR as described hereafter: an initial step to burst bacterial cells at 95°C for 30 s, followed by 45 cycles of denaturation at 95°C for 30 s, annealing at 55°C for 30 s, and extension at 72°C for 45 s, followed by a final extension step at 72°C for 5 min. PCR products were separated in 2% agarose gels, screened for positive insertion and sequenced by Beckman Genomics^®^.

*Data processing*. After removing the vector, sequences were aligned manually using the Seaview software [Gouy et al., 2010] and thoroughly investigated.

**SERRS-Hybridization assay**

*Oligonucleotide probes.* A SERRS 22-mer oligonucleotide labeled with one molecule of Rhodamine 6G (detection probe), a biotin labeled 21-mer oligonucleotide (capture probe) and 3 blocker oligonucleotides (29 to 31 bases) were purchased from Eurogentec^®^. The 21-mer biotin probe is modified with 3’-biotin that is linked to the oligonucleotide via the 9-atom spacer triethylene glycol. The 22-mer R6G-probe is modified with 5’-Rhodamine 6G (R6G), which is linked to the oligonucleotide via a C6 spacer. All DNA sequences used in this study are summarized in Table 1.

*Hybridization of target with blockers, capture and detection probes.* Hybridization was performed in a single step as follows. 10 μl of target DNA solution were mixed with 10 μl of the capture probe (10 μM), 10 μl of each detection probe (10 μM) and 10 μl of each blocking oligonucleotide (10^3^ times in excess compared to target DNA concentration). The blockers were initially prepared in 4xSSC, Tween 20 (0.5%). The resulting solution was heated to 95°C for 2 min to ensure denaturation of double-stranded DNA target, potential hairpins or autohybridized DNA. Hybridization was achieved in a thermocycler by lowering the temperature from 55°C to 25°C at a rate of 1°C per min.

*Immobilization.* Series of 25 μl of the stock solution of magnetic microbeads were rinsed three times in 25 μl of buffer 4xSSC, 0.5% Tween 20 using the magnetic particle concentrator (MPC). This step is called “activation” of the microbeads. Microbeads were re-suspended in 10 μl of buffer, and added to the hybridization solution for immobilization under gentle continuous stirring for 30 min at room temperature. The beads were finally washed twice in 150 μl of a 0.1xSSC, Tween 20 (0.5%) washing buffer using the MPC to remove the unbound material.

After resuspension in 60 μl of 0.1xSSC, 0.5% Tween 20 buffer, microbeads were finally heated at 95°C for 20 min for denaturation of both the DNA hybridized triplex and the biotin-streptavidin bound. The microbeads were then immobilized by the MPC and the supernatant was collected for SERRS measurements.

*SERRS measurements.* The silver colloid used for SERRS measurements was synthesized according to the Lee and Meisel protocol [Lee & Meisel, 1989]. 90 mg of silver nitrate AgNO_3_ were dissolved in 500 ml of distilled water and heated under continuous stirring until boiling. After addition of 10 ml of sodium citrate solution (1%) the solution was maintained at boiling for 90 min. The silver colloid has been stored in the dark at room temperature. All silver colloid aliquots used in this study came from the same batch.

The eluted R6G probes contained in the supernatant after the assay were analyzed following Feuillie *et al.* [2011]. 20 μl of the supernatant was mixed with 20 μl of spermine (10^-2^ mol.l^-1^) in a single-use PMMA spectroscopy cuvette. 500 μl of silver colloid and 500 μl of distilled water were added and the solution was then homogenized prior to SERRS measurement.

Samples were analyzed with a visible Horiba Jobin Yvon^®^ LabRam HR 800 Raman spectrometer, coupled to a Spectra Physics^®^ 2018 Ar^+^/Kr^+^ 24 laser tuned at 514.5 nm (LGL, ENS de Lyon). The laser power on the sample was adjusted between 1.5 and 2 mW. The spectra were acquired with a spectrometer grating of 600 gr.mm^-1^ centered at 1600 cm^-1^. Spectra result from 1x30 s accumulations.

**Sequence analysis**

First, among the 19 sequences obtained for the non-degraded double-stranded molecule N_5’_/N_3’_ (Suppl. Info., Table S1), 10/19 are perfect copies of the reference molecule, 8/19 display a single deletion and only 1/19 displays multiple deletions. Among the 8 one-base shorter molecules, 50% of the sequences display a single-base deletion at position 65 (Suppl. Info., Table S1), thus suggesting that the deletion is not randomly located. It actually reflects a common error occurring during the synthesis of oligonucleotides. Indeed, though the synthesis procedure leads mostly to the expected molecules, a few molecules with one missing base are also synthesized, and cannot be eliminated by purification (Eurogentec^®^, supplier communication). Second, for the molecules with one native strand N_5’_ or N_3’_ and one degraded strand (*i.e.* N_5’_/I_3’_, II_5’_/N_3’_, N_5’_/IV_3’_ and V_5’_/N_3’_, see Supplementary information), the reference N sequence is the most abundant obtained among the PCR products with 15/27, 16/27, 12/22 and 12/27 of the sequences obtained, respectively. The other sequences present short scale deletions from 1 to 17 bases and the average percentage of miscoding errors (0.14%) is 2.3 times larger than for the original molecule (Figure 3; Suppl. Info.).

**Table S1: Alignment of the sequences obtained from cloning-sequencing for the PCR amplification of the N_5’_/N_3’_ molecule.** The N molecule is taken as a reference. PCR amplification primers are removed from the analysis and appear only on the template molecule in red. Differences from the initial molecule are represented in red. For each haplotype sequence, the observed frequency and percentage are given.

|  |  | **5'3' Sequence** | **Freq.** | **%** |
| --- | --- | --- | --- | --- |
|  | **N** | **CATGAAGCACGCACACACCGCCCGTCACCCTCCTCAAGTGAATACAGGACACTCAAAACCTATTTAAACACACCAATCACACAAGAGGAGACAAGTCGTAACAAGGTAAGCATACTGGAAAGTGTGCTTGGACA** |  |  |
|  | **Consensus** | ............................................................................................... |  |  |
| N/N | >1 | ............................................................................................... | 10 | 53% |
|  | >2 | .........................................-..................................................... | 4 | 21% |
|  | >3 | .........................................................................-..................... | 1 | 5% |
|  | >4 | .............------............................................................................ | 1 | 5% |
|  | >5 | .....................-......................................................................... | 1 | 5% |
|  | >6 | ....................W-......................................................................... | 1 | 5% |
|  | >7 | .....................................................-......................................... | 1 | 5% |
| N/N - independant PCR results | PCR1 |  |  |  |
|  | >1 | ............................................................................................... | 3 | 75% |
|  | >3 | .........................................................................-..................... | 1 | 25% |
|  | PCR2 |  |  |  |
|  | >1 | ............................................................................................... | 2 | 67% |
|  | >2 | .........................................-..................................................... | 1 | 33% |
|  | PCR3 |  |  |  |
|  | >1 | ............................................................................................... | 3 | 50% |
|  | >2 | .........................................-..................................................... | 1 | 17% |
|  | >4 | .............------............................................................................ | 1 | 17% |
|  | >5 | .....................-......................................................................... | 1 | 17% |
|  | PCR4 |  |  |  |
|  | >1 | ............................................................................................... | 2 | 33% |
|  | >2 | .........................................-..................................................... | 2 | 33% |
|  | >6 | ....................W-......................................................................... | 1 | 17% |
|  | >7 | .....................................................-......................................... | 1 | 17% |

In each of the following tables, abasic sites are represented as red stars *. PCR amplification primers are removed from the analysis and appear only on the template molecule in red. Differences from the initial molecule are represented in red. For each haplotype sequence, the observed frequency and percentage are given.

|  | **Table S2: Alignment of the sequences obtained from cloning-sequencing for the PCR amplification of the N_5’_/I_3’_ molecule.** The N_5’_ and C_I_3’_ molecules are taken as a reference. The C_I_3’_ corresponds to I_3’_ strand in the reverse complementary orientation. | |  |  |
| --- | --- | --- | --- | --- |
|  |  | **5'3' Sequence** | **Freq.** | **%** |
|  | **N** | **CATGAAGCACGCACACACCGCCCGTCACCCTCCTCAAGTGAATACAGGACA-CTCAAAACCTATTTAAACACACCAATCACACAAGAGGAGACAAGTCGTAACAAGGTAAGCATACTGGAAAGTGTGCTTGGACA** |  |  |
|  | **C_I** | **CATGAAGCACGCACACACCGCCCGTCACCCTCCTCAAGTGAATAC*GGACA-CTCAAAACCTATTTAAACACACCAATCACACAAGAGGAGACAAGTCGTAACAAGGTAAGCATACTGGAAAGTGTGCTTGGACA** |  |  |
|  | **Consensus** | ..............................-................................................................. |  |  |
| N/I | >1 | ..............................-................................................................. | 15 | 56% |
|  | >2 | ..............................-.......................--........................................ | 1 | 4% |
|  | >3 | ..............................-..........................................................C.....G | 1 | 4% |
|  | >4 | ..............................-..................................................-.............. | 1 | 4% |
|  | >5 | ...................-..........-................................................................. | 1 | 4% |
|  | >6 | ..............................-.............................G................................... | 1 | 4% |
|  | >7 | ..............................-........................T........................................ | 1 | 4% |
|  | >8 | .........................------................................................................. | 1 | 4% |
|  | >9 | ..............................-.....-----------------........................................... | 1 | 4% |
|  | >10 | ..........-...................-....-............................................................ | 1 | 4% |
|  | >11 | ..............................-..........................................................T...... | 1 | 4% |
|  | >12 | .........----.................-................................................................. | 1 | 4% |
|  | >13 | ..............................C................................................................. | 1 | 4% |

Table S2 (suite)

| N/I - independant PCR results | PCR1 |  |  |  |
| --- | --- | --- | --- | --- |
|  | >1 | ................................................................................................ | 4 | 57% |
|  | >2 | ......................................................--........................................ | 1 | 14% |
|  | >3 | ..............................-..........................................................C.....G | 1 | 14% |
|  | >4 | ..............................-..................................................-.............. | 1 | 14% |
|  | PCR2 |  |  |  |
|  | >1 | ................................................................................................ | 4 | 57% |
|  | >5 | ...................-..........-................................................................. | 1 | 14% |
|  | >6 | ..............................-.............................G................................... | 1 | 14% |
|  | >7 | ..............................-........................T........................................ | 1 | 14% |
|  | PCR3 |  |  |  |
|  | >1 | ..............................-................................................................. | 2 | 29% |
|  | >8 | .........................------................................................................. | 1 | 14% |
|  | >9 | ..............................-.....-----------------........................................... | 1 | 14% |
|  | >10 | ..........-...................-....-............................................................ | 1 | 14% |
|  | >11 | ..............................-..........................................................T...... | 1 | 14% |
|  | >12 | .........----.................-................................................................. | 1 | 14% |
|  | PCR4 |  |  |  |
|  | >1 | ..............................-................................................................. | 5 | 83% |
|  | >13 | ..............................C................................................................. | 1 | 17% |

|  | **Table S3: Alignment of the sequences obtained from cloning-sequencing for the PCR amplification of the II_5’_/N_3’_ molecule.** The II_5’_ and C_N_3’_ molecules are taken as a reference. The C_N_3’_ corresponds to N_3’_ strand in the reverse complementary orientation. | |  |  |
| --- | --- | --- | --- | --- |
|  |  | **5'3' Sequence** | **Freq.** | **%** |
|  | **II** | **CATGAAGCACGCACACACCGCCCGTCACCCTCCTCAAGTGAATACAGGACACTCAAAACCT*TTTAAACAC*CCAATCACACAAGAGGAGACAAGTCGTAACAAGGTAAGCATACTGGAAAGTGTGCTTGGACA** |  |  |
|  | **C_N** | **CATGAAGCACGCACACACCGCCCGTCACCCTCCTCAAGTGAATACAGGACACTCAAAACCTATTTAAACACACCAATCACACAAGAGGAGACAAGTCGTAACAAGGTAAGCATACTGGAAAGTGTGCTTGGACA** |  |  |
|  | **Consensus** | ............................................................................................... |  |  |
| II/N | >1 | ............................................................................................... | 16 | 59% |
|  | >2 | ...................-........................................................................... | 1 | 4% |
|  | >3 | .....................-......................................................................... | 3 | 11% |
|  | >4 | ..............................................................................................G | 2 | 7% |
|  | >5 | .........................................-..................................................... | 3 | 11% |
|  | >6 | ..................................................................T............................ | 1 | 4% |
|  | >7 | ...........................................................................................A... | 1 | 4% |
| II/N - independant PCR results | PCR1 |  |  |  |
|  | >1 | ............................................................................................... | 7 | 100% |
|  | PCR2 |  |  |  |
|  | >1 | ............................................................................................... | 1 | 13% |
|  | >2 | ...................-........................................................................... | 1 | 13% |
|  | >3 | .....................-......................................................................... | 3 | 38% |
|  | >4 | ..............................................................................................G | 1 | 13% |
|  | >5 | .........................................-..................................................... | 2 | 25% |
|  | PCR3 |  |  |  |
|  | >1 | ............................................................................................... | 4 | 67% |
|  | >4 | ..............................................................................................G | 1 | 17% |
|  | >6 | ..................................................................T............................ | 1 | 17% |
|  | PCR4 |  |  |  |
|  | >1 | ............................................................................................... | 4 | 67% |
|  | >5 | .........................................-..................................................... | 1 | 17% |
|  | >7 | ...........................................................................................A... | 1 | 17% |
|  |  | |  |  |

|  | **Table S4: Alignment of the sequences obtained from cloning-sequencing for the PCR amplification of the N_5’_/IV_3’_ molecule.** The N_5’_ and C_IV_3’_ molecules are taken as a reference. The C_IV_3’_ corresponds to IV_3’_ strand in the reverse complementary orientation. | |  |  |
| --- | --- | --- | --- | --- |
|  |  | **5'3' Sequence** | **Freq.** | **%** |
|  | **N** | **CATGAAGCACGCACACACCGCCCGTCACCCTCCTCAAGTGAATACAGGACACTCAAAACCTATTTAAACACACCAATCACACAAGAGGAGACAAGTCGTAACAAGGTAAGCATACTGGAAAGTGTGCTTGGACA** |  |  |
|  | **C_IV** | **CATGAAGCACGCACACACCGCCCGTCACCCTCCTCAAGTGAATACA*GACACTCA*AACCTATT*AAACACAC*AATCACACAAGAGGAGACAAGTCGTAACAAGGTAAGCATACTGGAAAGTGTGCTTGGACA** |  |  |
|  | **Consensus** | ............................................................................................... |  |  |
| N/IV | >1 | ............................................................................................... | 12 | 55% |
|  | >2 | ............-.................................................................................. | 1 | 5% |
|  | >3 | ............................................-.................................................. | 1 | 5% |
|  | >4 | .......-.........................................T............................................. | 1 | 5% |
|  | >5 | ..................................................................--........................... | 1 | 5% |
|  | >6 | ......................................................................................-........ | 1 | 5% |
|  | >7 | ...........................W................................................................... | 1 | 5% |
|  | >8 | ...................-........................................................................... | 2 | 9% |
|  | >9 | ..............................................................................................- | 1 | 5% |
|  | >10 | ......--....................................................................................... | 1 | 5% |
|  | >1 | ............................................................................................... | 3 | 50% |
|  | >2 | ............-.................................................................................. | 1 | 17% |
|  | >3 | ............................................-.................................................. | 1 | 17% |
|  | >4 | .......-.........................................T............................................. | 1 | 17% |
|  | >1 | ............................................................................................... | 3 | 75% |
|  | >5 | ..................................................................--........................... | 1 | 25% |
|  | >1 | ............................................................................................... | 3 | 75% |
|  | >6 | ......................................................................................-........ | 1 | 25% |
|  | >1 | ............................................................................................... | 3 | 38% |
|  | >7 | ...........................W................................................................... | 1 | 13% |
|  | >8 | ...................-........................................................................... | 2 | 25% |
|  | >9 | ..............................................................................................- | 1 | 13% |
|  | >10 | ......--....................................................................................... | 1 | 13% |

|  | **Table S5: Alignment of the sequences obtained from cloning-sequencing for the PCR amplification of the V_5’_/N_3’_ molecule.** The V_5’_ and C_N_3’_ molecules are taken as a reference. The C_N_3’_ corresponds to N_3’_ strand in the reverse complementary orientation. | |  |  |
| --- | --- | --- | --- | --- |
|  |  | **5'3' Sequence** | **Freq.** | **%** |
|  | **V** | **CATGAAGCACGCACACACCGCCCGTCACCCTCCTCAAGTGAA*ACAGGACA*TCAAAACC*ATTTAAAC*CACCAATC*CACAAGAGGAGACAAGTCGTAACAAGGTAAGCATACTGGAAAGTGTGCTTGGACA** |  |  |
|  | **C_N** | **CATGAAGCACGCACACACCGCCCGTCACCCTCCTCAAGTGAATACAGGACACTCAAAACCTATTTAAACACACCAATCACACAAGAGGAGACAAGTCGTAACAAGGTAAGCATACTGGAAAGTGTGCTTGGACA** |  |  |
|  | **Consensus** | ............................................................................................... |  |  |
| V/N | >1 | ............................................................................................... | 12 | 44% |
|  | >2 | .............-................................................................................. | 1 | 4% |
|  | >3 | ..........................................................................-.................... | 1 | 4% |
|  | >4 | .................-............................................................................. | 1 | 4% |
|  | >5 | .......................G....................................................................... | 1 | 4% |
|  | >6 | .........-..................................................................................... | 1 | 4% |
|  | >7 | .....................-......................................................................... | 2 | 7% |
|  | >8 | ...-........................................................................................... | 2 | 7% |
|  | >9 | .........................................-..................................................... | 3 | 11% |
|  | >10 | .......................................-....................................................... | 1 | 4% |
|  | >11 | .........................................................................-..................... | 1 | 4% |
|  | >12 | .......................A....................................................................... | 1 | 4% |

Table S5 (suite)

| V/N - independant PCR results | PCR1 |  |  |  |
| --- | --- | --- | --- | --- |
|  | >1 | ............................................................................................... | 4 | 67% |
|  | >2 | .............-................................................................................. | 1 | 17% |
|  | >3 | ..........................................................................-.................... | 1 | 17% |
|  | PCR2 |  |  |  |
|  | >1 | ............................................................................................... | 2 | 29% |
|  | >4 | .................-............................................................................. | 1 | 14% |
|  | >5 | .......................G....................................................................... | 1 | 14% |
|  | >6 | .........-..................................................................................... | 1 | 14% |
|  | >7 | .....................-......................................................................... | 1 | 14% |
|  | >8 | ...-........................................................................................... | 1 | 14% |
|  | PCR3 |  |  |  |
|  | >1 | ............................................................................................... | 2 | 25% |
|  | >7 | .....................-......................................................................... | 1 | 13% |
|  | >9 | .........................................-..................................................... | 3 | 38% |
|  | >10 | .......................................-....................................................... | 1 | 13% |
|  | >11 | .........................................................................-..................... | 1 | 13% |
|  | PCR4 |  |  |  |
|  | >1 | ............................................................................................... | 4 | 67% |
|  | >8 | ...-........................................................................................... | 1 | 17% |
|  | >12 | .......................A....................................................................... | 1 | 17% |

|  | **Table S6: Alignment of the sequences obtained from cloning-sequencing for the PCR amplification of the I_5’_/I_3’_ molecule.** The I_5’_ and C_I_3’_ molecules are taken as a reference. The C_I_3’_ corresponds to I_3’_ strand in the reverse complementary orientation. | |  |  |
| --- | --- | --- | --- | --- |
|  |  | **5'3' Sequence** | **Freq.** | **%** |
|  | **I** | **CATGAAGCACGCACACACCGCCCGTCACCCTCCTCAAGTGAATACAGGACACTCAAAACCTATTTAAACAC*CCAATCACACAAGAGGAGACAAGTCGTAACAAGGTAAGCATACTGGAAAGTGTGCTTGGACA** |  |  |
|  | **C_I** | **CATGAAGCACGCACACACCGCCCGTCACCCTCCTCAAGTGAATAC*GGACACTCAAAACCTATTTAAACACACCAATCACACAAGAGGAGACAAGTCGTAACAAGGTAAGCATACTGGAAAGTGTGCTTGGACA** |  |  |
| I/I | >1 | ......................-----......................................................--------...... | 1 | 4% |
|  | >2 | ......-.................---------.............................................................. | 1 | 4% |
|  | >3 | .................................................--............................................ | 1 | 4% |
|  | >4 | ......................----------TC............................................................. | 1 | 4% |
|  | >5 | ............C.-----------------................................................................ | 1 | 4% |
|  | >6 | ........................C...................................................................... | 1 | 4% |
|  | >7 | ........................-.........G............................................................ | 1 | 4% |
|  | >8 | .............--------------------.............................................................. | 1 | 4% |
|  | >9 | ........................-........................--------------------------.................... | 1 | 4% |
|  | >10 | ........................-...................................................................... | 2 | 8% |
|  | >11 | ..............--------------................................................................... | 1 | 4% |
|  | >12 | ...............-----------------............................................................... | 1 | 4% |
|  | >13 | ..................................................-............................................ | 1 | 4% |
|  | >14 | ......................-------------............................................................ | 1 | 4% |
|  | >15 | .......................---..................................................................... | 1 | 4% |
|  | >16 | .................----------------.............................................................. | 1 | 4% |
|  | >17 | .....................-..C...................................................................... | 1 | 4% |
|  | >18 | .....................-------................................................................... | 1 | 4% |
|  | >19 | ........................................----------------....................................... | 1 | 4% |
|  | >20 | ..............-------------------.............................................................. | 1 | 4% |
|  | >21 | .................................................----.......................................... | 1 | 4% |
|  | >22 | ....................-------------.............................................................. | 1 | 4% |
|  | >23 | ......................-----------.............................................................. | 1 | 4% |
|  | >24 | ...........................--------------------------------.................................... | 1 | 4% |

Table S6 (suite)

| I/I - independant PCR results | PCR1 |  |  |  |
| --- | --- | --- | --- | --- |
|  | >1 | ......................-----......................................................--------...... | 1 | 14% |
|  | >2 | ......-.................---------.............................................................. | 1 | 14% |
|  | >3 | .................................................--............................................ | 1 | 14% |
|  | >4 | ......................----------TC............................................................. | 1 | 14% |
|  | >5 | ............C.-----------------................................................................ | 1 | 14% |
|  | >6 | ........................C...................................................................... | 1 | 14% |
|  | >7 | ........................-.........G............................................................ | 1 | 14% |
|  | PCR2 |  |  |  |
|  | >8 | .............--------------------.............................................................. | 1 | 14% |
|  | >9 | ........................-........................--------------------------.................... | 1 | 14% |
|  | >10 | ........................-...................................................................... | 2 | 29% |
|  | >11 | ..............--------------................................................................... | 1 | 14% |
|  | >12 | ...............-----------------............................................................... | 1 | 14% |
|  | >13 | ..................................................-............................................ | 1 | 14% |
|  | PCR3 |  |  |  |
|  | >14 | ......................-------------............................................................ | 1 | 17% |
|  | >15 | .......................---..................................................................... | 1 | 17% |
|  | >16 | .................----------------.............................................................. | 1 | 17% |
|  | >17 | .....................-..C...................................................................... | 1 | 17% |
|  | >18 | .....................-------................................................................... | 1 | 17% |
|  | >19 | ........................................----------------....................................... | 1 | 17% |
|  | PCR4 |  |  |  |
|  | >20 | ..............-------------------.............................................................. | 1 | 20% |
|  | >21 | .................................................----.......................................... | 1 | 20% |
|  | >22 | ....................-------------.............................................................. | 1 | 20% |
|  | >23 | ......................-----------.............................................................. | 1 | 20% |
|  | >24 | ...........................--------------------------------.................................... | 1 | 20% |

|  | **Table S7: Alignment of the sequences obtained from cloning-sequencing for the PCR amplification of the II_5’_/I_3’_ molecule.** The II_5’_ and C_I_3’_ molecules are taken as a reference. The C_I_3’_ corresponds to I_3’_ strand in the reverse complementary orientation. | |  |  |
| --- | --- | --- | --- | --- |
|  |  | **5'3' Sequence** | **Freq.** | **%** |
|  | **II** | **CATGAAGCACGCACACACCGCCCGTCACCCTCCTCAAGTGAATACAGGACACTCAAAACCT*TTTAAACAC*CCAATCACACAAGAGGAGACAAGTCGTAACAAGGTAAGCATACTGGAAAGTGTGCTTGGACA** |  |  |
|  | **C_I** | **CATGAAGCACGCACACACCGCCCGTCACCCTCCTCAAGTGAATAC*GGACACTCAAAACCTATTTAAACACACCAATCACACAAGAGGAGACAAGTCGTAACAAGGTAAGCATACTGGAAAGTGTGCTTGGACA** |  |  |
| II/I | >1 | .....................----------------------.................................................... | 1 | 5% |
|  | >2 | .......................------.................................................................. | 1 | 5% |
|  | >3 | ........................-----------............................................................ | 1 | 5% |
|  | >4 | .................----------.................-.................................................. | 1 | 5% |
|  | >5 | .....................--------------............................................................ | 1 | 5% |
|  | >6 | ......................------------............................................................. | 1 | 5% |
|  | >7 | ................-----------.................................................................... | 1 | 5% |
|  | >8 | ........................----................................................................... | 3 | 14% |
|  | >9 | ........................-...............................................................A...... | 1 | 5% |
|  | >10 | .........C.........-------------............................................................... | 1 | 5% |
|  | >11 | .......................----------.............................................................. | 1 | 5% |
|  | >12 | ........T...............C................................-------------....................--... | 1 | 5% |
|  | >13 | .......................-----................................................................... | 2 | 10% |
|  | >14 | .......................--...................................................................... | 1 | 5% |
|  | >15 | .................----------------.............................................................. | 1 | 5% |
|  | >16 | .....................--------..........................................T...............G....... | 1 | 5% |
|  | >17 | ..................---------------......................................-....................... | 1 | 5% |
|  | >18 | ........................-...................................................................... | 1 | 5% |

Table S7 (suite)

| II/I - independant PCR results | PCR1 |  |  |  |
| --- | --- | --- | --- | --- |
|  | >1 | .....................----------------------.................................................... | 1 | 17% |
|  | >2 | .......................------.................................................................. | 1 | 17% |
|  | >3 | ........................-----------............................................................ | 1 | 17% |
|  | >4 | .................----------.................-.................................................. | 1 | 17% |
|  | >5 | .....................--------------............................................................ | 1 | 17% |
|  | >6 | ......................------------............................................................. | 1 | 17% |
|  | PCR2 |  |  |  |
|  | >7 | ................-----------.................................................................... | 1 | 25% |
|  | >8 | ........................----................................................................... | 1 | 25% |
|  | >9 | ........................-...............................................................A...... | 1 | 25% |
|  | >10 | .........C.........-------------............................................................... | 1 | 25% |
|  | PCR3 |  |  |  |
|  | >8 | ........................----................................................................... | 2 | 50% |
|  | >11 | .......................----------.............................................................. | 1 | 25% |
|  | >12 | ........T...............C................................-------------....................--... | 1 | 25% |
|  | PCR4 |  |  |  |
|  | >13 | .......................-----................................................................... | 2 | 29% |
|  | >14 | .......................--...................................................................... | 1 | 14% |
|  | >15 | .................----------------.............................................................. | 1 | 14% |
|  | >16 | .....................--------..........................................T...............G....... | 1 | 14% |
|  | >17 | ..................---------------......................................-....................... | 1 | 14% |
|  | >18 | ........................-...................................................................... | 1 | 14% |

|  | **Table S8: Alignment of the sequences obtained from cloning-sequencing for the PCR amplification of the I_5’_/IV_3’_ molecule.** The I_5’_ and C_IV_3’_ molecules are taken as a reference. The C_IV_3’_ corresponds to IV_3’_ strand in the reverse complementary orientation. | |  |  |
| --- | --- | --- | --- | --- |
|  |  | **5'3' Sequence** | **Freq.** | **%** |
|  | **I** | **CATGAAGCACGCACACACCGCCCGTCAC-CCTCCTCAAGTGAATACAGGACACTCAAAACCTATTTAAACAC*CCAATCACACAAGAGGAGACAAGTCGTAACAAGGTAAGCATACTGGAAAGTGTGCTTGGACA** |  |  |
|  | **C_IV** | **CATGAAGCACGCACACACCGCCCGTCAC-CCTCCTCAAGTGAATACA*GACACTCA*AACCTATT*AAACACAC*AATCACACAAGAGGAGACAAGTCGTAACAAGGTAAGCATACTGGAAAGTGTGCTTGGACA** |  |  |
| I/IV | >1 | --.....-...........................................T............................................ | 1 | 5% |
|  | >2 | .......-...........................................C.-.......................................... | 5 | 23% |
|  | >3 | .......-................................-------------........................................... | 1 | 5% |
|  | >4 | .......-.....................-----------------------------.......................-.............. | 1 | 5% |
|  | >5 | .......-...........................................T............................................ | 1 | 5% |
|  | >6 | .......C...........................................C.-.......................................... | 1 | 5% |
|  | >7 | .......-..--------------------------------------------------------------........................ | 1 | 5% |
|  | >8 | ..A....-----------------------------------------------------.................................... | 1 | 5% |
|  | >9 | .......-.........................................-----.......................................... | 1 | 5% |
|  | >10 | .......-..........................................AT.------............-........................ | 1 | 5% |
|  | >11 | .......-.......................................-...C.-.......................................... | 1 | 5% |
|  | >12 | .......-....................................--------------------................................ | 1 | 5% |
|  | >13 | .......-...----------------------------------------------------------........................... | 1 | 5% |
|  | >14 | .......-...........................................------------------........................... | 1 | 5% |
|  | >15 | .......-......................................-----------------................................. | 1 | 5% |
|  | >16 | ....T..-...........................-----------------.............G.............................. | 1 | 5% |
|  | >17 | .......-..................................---------------------................................. | 1 | 5% |
|  | >18 | .......-...........................................-------------------------.................... | 1 | 5% |

Table S8 (suite)

| I/IV - independant PCR results | PCR1 |  |  |  |
| --- | --- | --- | --- | --- |
|  | >1 | --.....-...........................................T............................................ | 1 | 17% |
|  | >2 | .......-...........................................C.-.......................................... | 2 | 33% |
|  | >3 | .......-................................-------------........................................... | 1 | 17% |
|  | >4 | .......-.....................-----------------------------.......................-.............. | 1 | 17% |
|  | >5 | .......-...........................................T............................................ | 1 | 17% |
|  | PCR2 |  |  |  |
|  | >2 | .......-...........................................C.-.......................................... | 2 | 33% |
|  | >6 | .......C...........................................C.-.......................................... | 1 | 17% |
|  | >7 | .......-..--------------------------------------------------------------........................ | 1 | 17% |
|  | >8 | ..A....-----------------------------------------------------.................................... | 1 | 17% |
|  | >9 | .......-.........................................-----.......................................... | 1 | 17% |
|  | PCR3 |  |  |  |
|  | >2 | .......-...........................................C.-.......................................... | 1 | 17% |
|  | >10 | .......-..........................................AT.------............-........................ | 1 | 17% |
|  | >11 | .......-.......................................-...C.-.......................................... | 1 | 17% |
|  | >12 | .......-....................................--------------------................................ | 1 | 17% |
|  | >13 | .......-...----------------------------------------------------------........................... | 1 | 17% |
|  | >14 | .......-...........................................------------------........................... | 1 | 17% |
|  | PCR4 |  |  |  |
|  | >15 | .......-......................................-----------------................................. | 1 | 25% |
|  | >16 | ....T..-...........................-----------------.............G.............................. | 1 | 25% |
|  | >17 | .......-..................................---------------------................................. | 1 | 25% |
|  | >18 | .......-...........................................-------------------------.................... | 1 | 25% |

|  | **Table S9: Alignment of the sequences obtained from cloning-sequencing for the PCR amplification of the V_5’_/I_3’_ molecule.** The V_5’_ and C_I_3’_ molecules are taken as a reference. The C_I_3’_ corresponds to I_3’_ strand in the reverse complementary orientation. | |  |  |
| --- | --- | --- | --- | --- |
|  |  | **5'3' Sequence** | **Freq.** | **%** |
|  | **V** | **CATGAAGCACGCACACACCGCCCGTCACCCTCCTCAAGTGAA*ACAGGACA*TCAAAACC*ATTTAAAC*CACCAATC*CACAAGAGGAGACAAGTCGTAACAAGGTAAGCATACTGGAAAGTGTGCTTGGACA** |  |  |
|  | **C_I** | **CATGAAGCACGCACACACCGCCCGTCACCCTCCTCAAGTGAATAC*GGACACTCAAAACCTATTTAAACACACCAATCACACAAGAGGAGACAAGTCGTAACAAGGTAAGCATACTGGAAAGTGTGCTTGGACA** |  |  |
| V/I | >1 | ........................--..................................................................... | 1 | 5% |
|  | >2 | ........................-...................................................................... | 2 | 11% |
|  | >3 | .......................----.................................................................... | 2 | 11% |
|  | >4 | ...........--------------------------.......................................................... | 1 | 5% |
|  | >5 | ........................-----.................................................................. | 1 | 5% |
|  | >6 | ...........----------------------.............................................................. | 1 | 5% |
|  | >7 | ........................--------............................................................... | 1 | 5% |
|  | >8 | .........-------------------......................-.................A.......................... | 1 | 5% |
|  | >9 | ....--.................----------.............................................................. | 1 | 5% |
|  | >10 | .......................--...................................................................... | 1 | 5% |
|  | >11 | ....................-----C.G......................................................---.......... | 1 | 5% |
|  | >12 | ......................--------................................................................. | 1 | 5% |
|  | >13 | ..........................................-------.............................................. | 1 | 5% |
|  | >14 | ...C....................-...................................................................... | 1 | 5% |
|  | >15 | ........................C............-......................................................... | 1 | 5% |
|  | >16 | ........................--------------------................................................... | 1 | 5% |
|  | >17 | ...-.............-------------------........................................................... | 1 | 5% |

Table S9 (suite)

| V/I - independant PCR results | PCR1 |  |  |  |
| --- | --- | --- | --- | --- |
|  | >1 | ........................--..................................................................... | 1 | 25% |
|  | >2 | ........................-...................................................................... | 2 | 50% |
|  | >3 | .......................----.................................................................... | 1 | 25% |
|  | PCR2 |  |  |  |
|  | >4 | ...........--------------------------.......................................................... | 1 | 17% |
|  | >5 | ........................-----.................................................................. | 1 | 17% |
|  | >6 | ...........----------------------.............................................................. | 1 | 17% |
|  | >7 | ........................--------............................................................... | 1 | 17% |
|  | >8 | .........-------------------......................-.................A.......................... | 1 | 17% |
|  | >9 | ....--.................----------.............................................................. | 1 | 17% |
|  | PCR3 |  |  |  |
|  | >3 | .......................----.................................................................... | 1 | 20% |
|  | >10 | .......................--...................................................................... | 1 | 20% |
|  | >11 | ....................-----C.G......................................................---.......... | 1 | 20% |
|  | >12 | ......................--------................................................................. | 1 | 20% |
|  | >13 | ..........................................-------.............................................. | 1 | 20% |
|  | PCR4 |  |  |  |
|  | >14 | ...C....................-...................................................................... | 1 | 25% |
|  | >15 | ........................C............-......................................................... | 1 | 25% |
|  | >16 | ........................--------------------................................................... | 1 | 25% |
|  | >17 | ...-.............-------------------........................................................... | 1 | 25% |

|  | **Table S10: Alignment of the sequences obtained from cloning-sequencing for the PCR amplification of the II_5’_/IV_3’_ molecule.** The II_5’_ and C_IV_3’_ molecules are taken as a reference. The C_IV_3’_ corresponds to IV_3’_ strand in the reverse complementary orientation. | |  |  |
| --- | --- | --- | --- | --- |
|  |  | **5'3' Sequence** | **Freq.** | **%** |
|  | **II** | **CATGAAGCACGCACACACCGCCCGTCACCCTCCTCAAGTGAATACAGGACACTCAAAACCT*TTTAAACAC*CCAATCACACAAGAGGAGACAAGTCGTAACAAGGTAAGCATACTGGAAAGTGTGCTTGGACA** |  |  |
|  | **C_IV** | **CATGAAGCACGCACACACCGCCCGTCACCCTCCTCAAGTGAATACA*GACACTCA*AACCTATT*AAACACAC*AATCACACAAGAGGAGACAAGTCGTAACAAGGTAAGCATACTGGAAAGTGTGCTTGGACA** |  |  |
| II/IV | >1 | .........................-.........------------------------------------.............A.......... | 1 | 4% |
|  | >2 | ...................................-----------------------.G................................... | 1 | 4% |
|  | >3 | ......-...............................---------------------------------------------------...... | 1 | 4% |
|  | >4 | ............................----------------------------------------........................... | 2 | 8% |
|  | >5 | ........................----------------------------........................................... | 1 | 4% |
|  | >6 | .......................................----------------------------------...................... | 1 | 4% |
|  | >7 | ....----------------------------------------------------------------------------------......... | 1 | 4% |
|  | >8 | ..............-------------------------------------------------------------------------------.. | 1 | 4% |
|  | >9 | ........................-.............................-----------------....-----............... | 1 | 4% |
|  | >10 | T....----------------------------------------------------------------------------------------.. | 1 | 4% |
|  | >11 | ..............-----------------------------------------........................................ | 1 | 4% |
|  | >12 | .........................A..........----------------------------............................... | 1 | 4% |
|  | >13 | ....................................T---------------------------------------------------------- | 1 | 4% |
|  | >14 | ......................................----------------------................................... | 1 | 4% |
|  | >15 | ...................................-----------------........................................... | 1 | 4% |
|  | >16 | .....................................--------------............................................ | 1 | 4% |
|  | >17 | ....................--------------------------------------------------......................... | 1 | 4% |
|  | >18 | ...................----------------------------------------------------------.................. | 1 | 4% |
|  | >19 | ............................--------------------------------------------------------------..... | 1 | 4% |
|  | >20 | ..............................----------------------------------------------................... | 1 | 4% |
|  | >21 | .................-------------------------------------.A....................................... | 1 | 4% |
|  | >22 | .............................----------------------............................................ | 1 | 4% |
|  | >23 | .----------------------------------------------------------------------------.................. | 1 | 4% |

Table S10 (suite)

| II/IV - independant PCR results | PCR1 |  |  |  |
| --- | --- | --- | --- | --- |
|  | >1 | .........................-.........------------------------------------.............A.......... | 1 | 17% |
|  | >2 | ...................................-----------------------.G................................... | 1 | 17% |
|  | >3 | ......-...............................---------------------------------------------------...... | 1 | 17% |
|  | >4 | ............................----------------------------------------........................... | 1 | 17% |
|  | >5 | ........................----------------------------........................................... | 1 | 17% |
|  | >6 | .......................................----------------------------------...................... | 1 | 17% |
|  | PCR2 |  |  |  |
|  | >7 | ....----------------------------------------------------------------------------------......... | 1 | 20% |
|  | >8 | ..............-------------------------------------------------------------------------------.. | 1 | 20% |
|  | >9 | ........................-.............................-----------------....-----............... | 1 | 20% |
|  | >10 | T....----------------------------------------------------------------------------------------.. | 1 | 20% |
|  | >11 | ..............-----------------------------------------........................................ | 1 | 20% |
|  | PCR3 |  |  |  |
|  | >4 | ............................----------------------------------------........................... | 1 | 17% |
|  | >12 | .........................A..........----------------------------............................... | 1 | 17% |
|  | >13 | ....................................T---------------------------------------------------------- | 1 | 17% |
|  | >14 | ......................................----------------------................................... | 1 | 17% |
|  | >15 | ...................................-----------------........................................... | 1 | 17% |
|  | >16 | .....................................--------------............................................ | 1 | 17% |
|  | PCR4 |  |  |  |
|  | >17 | ....................--------------------------------------------------......................... | 1 | 14% |
|  | >18 | ...................----------------------------------------------------------.................. | 1 | 14% |
|  | >19 | ............................--------------------------------------------------------------..... | 1 | 14% |
|  | >20 | ..............................----------------------------------------------................... | 1 | 14% |
|  | >21 | .................-------------------------------------.A....................................... | 1 | 14% |
|  | >22 | .............................----------------------............................................ | 1 | 14% |
|  | >23 | .----------------------------------------------------------------------------.................. | 1 | 14% |

|  | **Table S11: Alignment of the sequences obtained from cloning-sequencing for the PCR amplification of the V_5’_/IV_3’_ molecule.** The V_5’_ and C_IV_3’_ molecules are taken as a reference. The C_IV_3’_ corresponds to IV_3’_ strand in the reverse complementary orientation. | |  |  |
| --- | --- | --- | --- | --- |
|  |  | **5'3' Sequence** | **Freq.** | **%** |
|  | **V** | **CATGAAGCACGCACACACCGCCCGTCACCCTCCTCAAGTGAA*ACAGGACA*TCAAAACC*ATTTAAAC*CACCAATC*CACAAGAGGAGACAAGTCGTAACAAGGTAAGCATACTGGAAAGTGTGCTTGGACA** |  |  |
|  | **C_IV** | **CATGAAGCACGCACACACCGCCCGTCACCCTCCTCAAGTGAATACA*GACACTCA*AACCTATT*AAACACAC*AATCACACAAGAGGAGACAAGTCGTAACAAGGTAAGCATACTGGAAAGTGTGCTTGGACA** |  |  |
| V/IV | >1 | ......................-----------------------------------...................................... | 3 | 13% |
|  | >2 | ............GT..----------------------------------------------------------------......-........ | 1 | 4% |
|  | >3 | ........................------------------------------......................................... | 1 | 4% |
|  | >4 | ..----------------------------------------------------......................................... | 1 | 4% |
|  | >5 | T........-----------------------------------------------....................................... | 1 | 4% |
|  | >6 | ...----------------------------------------------------------------............................ | 1 | 4% |
|  | >7 | .........--------------------------------------------------.................................... | 1 | 4% |
|  | >8 | .................----------------------------------------------------------------------------.. | 2 | 9% |
|  | >9 | --------------------------------------------------------------................................. | 1 | 4% |
|  | >10 | ........................----------------------------------------------------------............. | 1 | 4% |
|  | >11 | ----------------------------------------------------------..................................... | 1 | 4% |
|  | >12 | ..........-------------------------------------------------------------------.................. | 1 | 4% |
|  | >13 | ....----------------------------------------------------------------........................... | 1 | 4% |
|  | >14 | ---------------------------------------------------------------------------------------------.. | 1 | 4% |
|  | >15 | ..-----------------------------------------------------------------............................ | 1 | 4% |
|  | >16 | ..............-------------------------------------------------------------------------------T. | 1 | 4% |
|  | >17 | ......T-------------------------------------------------------------------------------------... | 1 | 4% |
|  | >18 | .........................----------------------------.......................................... | 1 | 4% |
|  | >19 | ....----------------------------------------------------------------------------------------... | 1 | 4% |
|  | >20 | ....................TA----------------------------------....................................... | 1 | 4% |

Table S11 (suite)

| V/IV - independant PCR results | PCR1 |  |  |  |
| --- | --- | --- | --- | --- |
|  | >1 | ......................-----------------------------------...................................... | 2 | 33% |
|  | >2 | ............GT..----------------------------------------------------------------......-........ | 1 | 17% |
|  | >3 | ........................------------------------------......................................... | 1 | 17% |
|  | >4 | ..----------------------------------------------------......................................... | 1 | 17% |
|  | >5 | T........-----------------------------------------------....................................... | 1 | 17% |
|  | PCR2 |  |  |  |
|  | >6 | ...----------------------------------------------------------------............................ | 1 | 17% |
|  | >7 | .........--------------------------------------------------.................................... | 1 | 17% |
|  | >8 | .................----------------------------------------------------------------------------.. | 1 | 17% |
|  | >9 | --------------------------------------------------------------................................. | 1 | 17% |
|  | >10 | ........................----------------------------------------------------------............. | 1 | 17% |
|  | >11 | ----------------------------------------------------------..................................... | 1 | 17% |
|  | PCR3 |  |  |  |
|  | >1 | ......................-----------------------------------...................................... | 1 | 25% |
|  | >12 | ..........-------------------------------------------------------------------.................. | 1 | 25% |
|  | >13 | ....----------------------------------------------------------------........................... | 1 | 25% |
|  | >14 | ---------------------------------------------------------------------------------------------.. | 1 | 25% |
|  | PCR4 |  |  |  |
|  | >8 | .................----------------------------------------------------------------------------.. | 1 | 14% |
|  | >15 | ..-----------------------------------------------------------------............................ | 1 | 14% |
|  | >16 | ..............-------------------------------------------------------------------------------T. | 1 | 14% |
|  | >17 | ......T-------------------------------------------------------------------------------------... | 1 | 14% |
|  | >18 | .........................----------------------------.......................................... | 1 | 14% |
|  | >19 | ....----------------------------------------------------------------------------------------... | 1 | 14% |
|  | >20 | ....................TA----------------------------------....................................... | 1 | 14% |
